# A HML6 endogenous retrovirus on chromosome 3 is upregulated in amyotrophic lateral sclerosis motor cortex

**DOI:** 10.1038/s41598-021-93742-3

**Published:** 2021-07-12

**Authors:** Ashley R. Jones, Alfredo Iacoangeli, Brett N. Adey, Harry Bowles, Aleksey Shatunov, Claire Troakes, Jeremy A. Garson, Adele L. McCormick, Ammar Al-Chalabi

**Affiliations:** 1 Department of Basic and Clinical Neuroscience, Maurice Wohl Clinical Neuroscience Institute, Institute of Psychiatry, Psychology and Neuroscience, King’s College London, London, SE5 9NU UK; 2 Department of Biostatistics and Health Informatics, Institute of Psychiatry, Psychology and Neuroscience, King’s College London, London, UK; 3 National Institute for Health Research Biomedical Research Centre and Dementia Unit at South London and Maudsley NHS Foundation Trust and King’s College London, London, UK; 4 Social Genetic and Developmental Psychiatry Centre, Institute of Psychiatry, Psychology and Neuroscience, King’s College London, London, UK; 5 NIHR Maudsley Biomedical Research Centre, South London and Maudsley NHS Trust, King’s College London, London, UK; 6 National Institute for Health Research Biomedical Research Centre at Guy’s and St Thomas’ NHS Foundation Trust and King’s College London, London, UK; 7 MRC London Neurodegenerative Diseases Brain Bank, Institute of Psychiatry, Psychology and Neuroscience, King’s College London, London, UK; 8 Division of Infection and Immunity, University College London, London, UK; 9 School of Life Sciences, University of Westminster, London, UK

**Keywords:** RNA sequencing, Motor neuron disease, Retrovirus, HIV infections, Brain

## Abstract

There is increasing evidence that endogenous retroviruses (ERVs) play a significant role in central nervous system diseases, including amyotrophic lateral sclerosis (ALS). Studies of ALS have consistently identified retroviral enzyme reverse transcriptase activity in patients. Evidence indicates that ERVs are the cause of reverse transcriptase activity in ALS, but it is currently unclear whether this is due to a specific ERV locus or a family of ERVs. We employed a combination of bioinformatic methods to identify whether specific ERVs or ERV families are associated with ALS. Using the largest *post-mortem* RNA-sequence datasets available we selectively identified ERVs that closely resembled full-length proviruses. In the discovery dataset there was one ERV locus (HML6_3p21.31c) that showed significant increased expression in *post-mortem* motor cortex tissue after multiple-testing correction. Using six replication *post-mortem* datasets we found HML6_3p21.31c was consistently upregulated in ALS in motor cortex and cerebellum tissue. In addition, HML6_3p21.31c showed significant co-expression with cytokine binding and genes involved in EBV, HTLV-1 and HIV type-1 infections. There were no significant differences in ERV family expression between ALS and controls. Our results support the hypothesis that specific ERV loci are involved in ALS pathology.

## Introduction

Amyotrophic lateral sclerosis (ALS) is a neurodegenerative disease of cortical, bulbar and spinal motor neurons resulting in progressive weakness and ultimately death, typically within 3–5 years, as a result of neuromuscular respiratory failure. Genetic risk factors have been identified: up to 10% of people with apparently sporadic ALS have a pathologically expanded hexanucleotide repeat in the gene *C9orf72*^1,2^; mutations in the genes *TARDBP*, *FUS* and *SOD1* account for a further 4–5%. For most, the cause of the disease is unknown.

Our incomplete understanding of the causes of ALS is reflected in two major obstacles to progress. The first is that only a small proportion of the measured heritability of ALS is accounted for by observed genetic variation. While advances in modelling polygenicity and rare DNA variants may explain some of this missing heritability in the future, other genomic elements, not captured using genome-wide association (GWAS) and whole genome sequencing (WGS) studies, play a role in disease^3,4^. Among such genomic features are mobile genetic elements (including endogenous retroviruses)^3,5^, DNA structural variation^4^, and epigenetics^6^.

The second obstacle is that there is a significant non-genetic component that contributes to the risk of ALS^7–9^, and despite concerted efforts there is little consensus or replicated evidence as to what this is.

Research into several neurological diseases has shown that human endogenous retroviruses (ERVs) play a significant role in aetiology^10,11^. ERVs are retroviruses that have integrated into genomic DNA and become components of the mammalian genome over millions of years of evolution. It is estimated that 8–9% of the human genome consists of ERVs in comparison to the 1–2% comprising protein-coding genes. Most ERVs are unable to transcribe, translate or replicate, due to the accumulation of mutations in their DNA sequence. However, a significant proportion still retain full-length sequences that encode retroviral proteins. Furthermore, ERVs can regulate the expression of genes nearby through genetic components such as non-coding RNAs, and enhancer and promotor regions that include long terminal repeats (LTRs) that contain TATA-box promoters and AATAAA motifs for polyadenylation^12,13^.

Aberrant ERV transcription and protein expression have been implicated in several central nervous system diseases, including multiple sclerosis (MS)^14–16^, Alzheimer’s disease (AD)^17,18^, schizophrenia^19–21^, chronic inflammatory demyelinating polyneuropathy^17^ and ALS^22–26^. ERVs can be regulated by DNA polymorphisms, variation in methylation and chromatin state, and transactivation. Viral transactivation of ERV expression by exogenous viruses can have consequences on disease susceptibility and progression, such as changes in HERV-W expression due to herpes simplex and Epstein–Barr viruses in MS^15,27–32^ and changes in HERV-K expression in response to HIV Type 1^33–35^. There has been renewed interest in the role exogenous viruses in neurodegeneration, with findings implicating herpes viruses in AD^36–38^.

Over the last 20 years, a number of studies have identified a signature of retrovirus expression in the form of increased reverse transcriptase activity in ALS serum samples^22–24,39^. There is elevated HERV-K RNA and protein expression in *post-mortem* ALS brain in comparison with the levels detected in healthy and disease-related controls^25,40^, although this is not confirmed in all studies^41,42^. A key finding is that HERV-K colocalises, coregulates and binds with TDP-43^25,40^, a protein that shows intracellular aggregation in up to 95% of people with ALS, *post-mortem*. In vivo and in vitro models indicate that upregulation of ERVs, as a consequence of TDP-43 overexpression, contribute to a more severe form^43^ which is likely to be cell-specific^44^. However, it remains unclear whether the association of ERVs with ALS is due to a single ERV locus, a specific ERV family or due to multiple ERV families.

We therefore applied a combination of bioinformatic methods to multiple *post-mortem* ALS cohorts to test the hypothesis that specific ERVs or ERV families are associated with ALS. If confirmed, this would allow the development of treatments specifically targeting this group of retroviruses.

## Results

### ERV locus HML6_3p21.31c is differentially expressed in ALS

We used the curated ERVMap database and protocol to test for differentially expressed ERVs comparing ALS (n = 80) and controls (n = 28) using RNA-sequence data from King’s College London (primary motor cortex); see Table 1. 1654 of 3237 ERV transcripts had sufficient read-counts (> 10) across samples for testing. ERVs previously associated with ALS but not included in ERVMap were analysed independently (see Supplementary Table S1).

**Table 1:** Summary of sample characteristics across datasets.

Disease status	N	Male:Female ratio	Mean age of death	Mean PMD	Mean RIN
**KCL**					
*Motor cortex (primary)*					
Cases	80	43:37	67.46	37.09	5.27
Controls	28	15:13	66.17	26.12	6.36
**GSE137810**					
*Motor cortex (lateral)*					
Cases	32	16:16	63.83	10.02	6.68
Controls	6	3:3	63.98	11.41	6.33
*Motor cortex (medial)*					
Cases	32	16:16	67.43	9.43	6.8
Controls	5	3:2	68.4	11.1	6.5
*Frontal cortex (various)*					
Cases	76	31:45	NA	NA	NA
Controls	7	3:4	NA	NA	NA
*Cerebellum*					
Cases	52	25:27	70.3	NA	NA
Controls	5	1:4	71	NA	NA
**GSE67196**					
*Prefrontal cortex*					
Cases	14	7:7	51.59	10.21	NA
Controls	7	4:3	69.25	15.86	NA
*Cerebellum*					
Cases	14	7:7	51.59	10.21	NA
Controls	7	4:3	7.58	15.14	NA

*PMD* post-mortem delay, *RIN* RNA integrity number.

**Figure 1:**
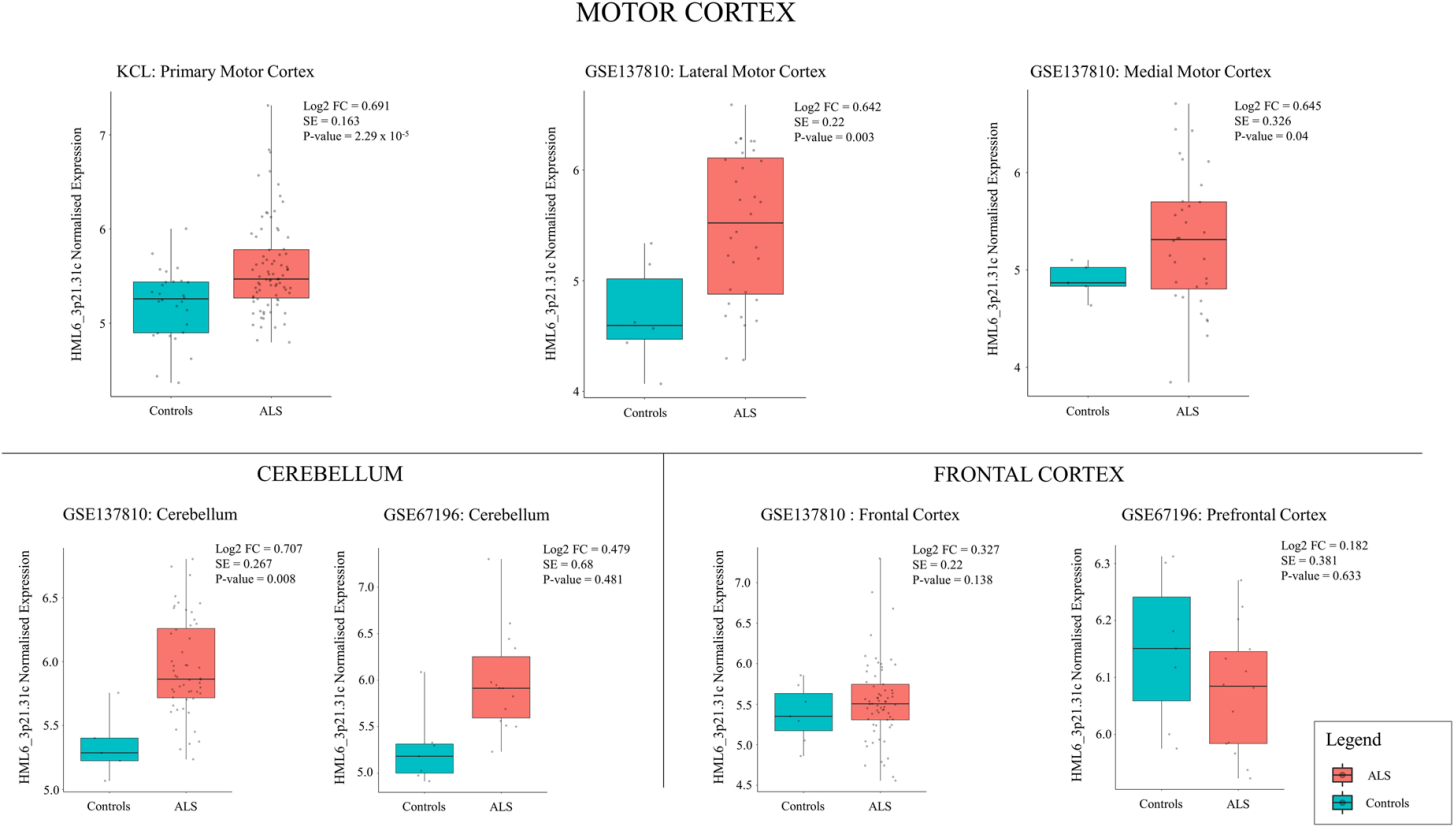
Jitter boxplots comparing HML6_3p21.31c expression between ALS and controls across seven *post-mortem* RNA-sequence datasets. We found increased expression of HML6_3p21.31c in all motor cortex and cerebellum cohorts, but not in the frontal cortex tissue. *X-axes:* Disease status. *Y-axes*: Normalised expression of HML6_3p21.31c. *Dots*: HML6_3p21.31c expression for each sample. *Boxplot*: Boxplot of the distribution of HML6_3p21.31c. *Boxplot colour*: Disease status (ALS: Red; Control: Blue). *Figure titles*: Data source and tissue. Log2 FC: Log2 Fold-change, SE: Standard error, *p* value: Derived from differential expression analysis.

One ERV (HML6_3p21.31c) showed a significant increase in expression in ALS with respect to age-sex matched non-ALS controls after correcting for multiple testing (log2 fold-change = 0.691, standard error = 0.163, *p* value = 2.29 × 10^−5^, adjusted *p* value = 0.03). On human genome assembly 38 (hg38), HML6_3p21.31c is located at chr3:46426676–46433564 (see Fig. 1, Table 2, and Supplementary Table S2a).

**Table 2:**
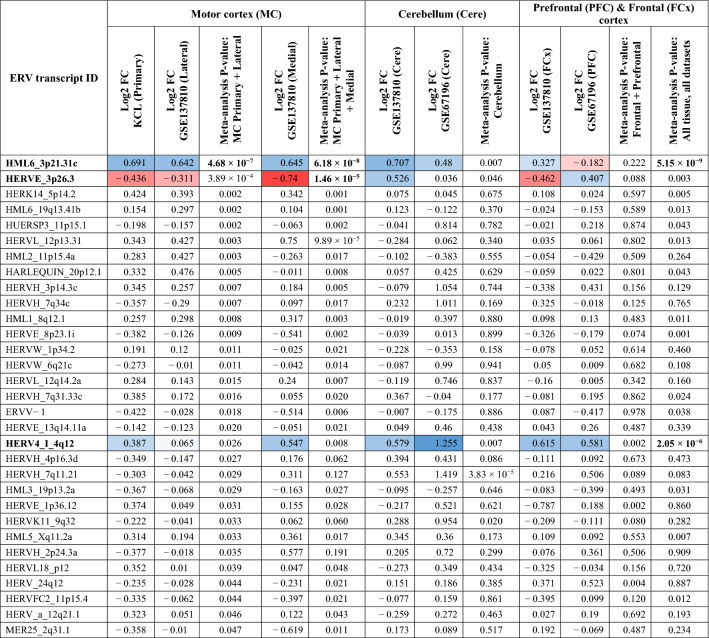
Differential expression analyses of retrovirus transcripts.

This table displays log2 fold-change between ALS cases and controls for each tissue, and the *p* value from the Stouffer’s meta-analysis. Transcripts are shown if the Stouffer’s meta-analysis had *p* < 0.05, incorporating the KCL primary motor cortex dataset and the TA lateral motor cortex dataset.

Bold: Significant Stouffer’s meta-analysis *p* value, beyond Bonferroni multiple-testing correction for 3237 transcripts (*p* < 1.5 × 10^−5^). Cells highlighted in blue indicate increased expression in ALS samples; cells highlighted in red indicate decreased expression in ALS samples, compared to controls, where transcripts had a *p* < 1.5 × 10^−5^ in any meta-analysis.

*KCL* King’s College London, *MC* motor cortex, *Cere* cerebellum, *FCx* frontal cortex (various), *PFC* prefrontal cortex, *Lat* lateral, *Med* medial, *FC* fold-change.

We then performed the same analysis using lateral motor cortex taken from the GSE137810 dataset (ALS n = 32, Controls n = 6). HML6_3p21.31c showed a significant increase in expression in ALS with a similar fold-change to the KCL cohort (log2 fold-change = 0.642, standard error = 0.220, *p* value = 0.003). See Fig. 1, Table 2, and Supplementary Table S2a.

To test if other ERV transcripts were significantly differentially expressed across both King’s College London and GSE137810 datasets we performed a Stouffer’s meta-analysis, with log2 fold-change as effect direction and sample size used as weights. Only HML6_3p21.31c was significant after correcting for multiple-testing, with Stouffer’s Z = 5.039, *p* value = 4.675 × 10^−7^ (Bonferroni threshold = 3.546 × 10^−5^). See Table 2 and Supplementary Table S2a.

To test if HML6_3p21.31c was significantly expressed in other brain areas we performed the same analysis in medial motor cortex, cerebellum and across frontal cortex regions using RNA-seq data from GSE137810 and GSE67196. We found a similar relationship between HML6_3p21.31c and ALS in the medial motor cortex (log2 fold-change = 0.645, standard error = 0.326, *p* value = 0.04). In addition, using Stouffer’s method, we found significant increased expression in cerebellum tissue using GSE137810 and GSE67196 datasets (Z = 2.721, *p* value = 0.006) but not in frontal regions (Z = 1.22, *p* value = 0.22). Please note that there are overlapping samples across cerebellum and prefrontal cortex datasets. See Fig. 1, Table 2, and Supplementary Table S2b,c.

The HML6 ERV family in general, modelled as a collapsed transcript element using TETranscripts, did not show significantly increased expression in ALS (see Supplementary Table S3). Previous research has identified specific ERV loci in the HML2 family as showing differential expression in ALS^40^. We did not find differential expression of these loci in motor cortex, cerebellum or prefrontal cortex. HML2 as a family of ERVs did not show differential expression between ALS and controls across tissue and studies (see Supplementary Table S3).

To assess whether differences in expression could be driven by differences in cell composition between ALS and control donors, we used the BRETIGEA^45^ cell marker database to estimate relative proportions of neurons across KCL samples. We did not find significant differences in estimated neuron proportions between ALS and controls, while controlling for covariates gender, age, *post-mortem* delay, RIN and surrogate variables (beta = 0.035, standard error = 0.023, *p* value = 0.150).

### ERV locus HML6_3p21.31c co-expresses with genes involved in cytokine pathways and HIV

To assess the functional involvement of HML6_3p21.31c in *post-mortem* ALS we performed weighted co-expression network analysis integrating gene expression with ERV expression. Our network analyses included both ALS cases and controls. We performed network analyses using the KCL primary motor cortex cohort as well as the GSE137810 lateral motor cortex.

**Figure 2:**
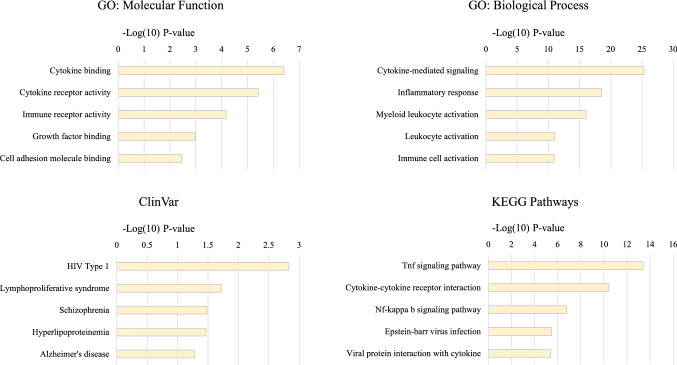
Gene function enrichment analyses of yellow network-module. This analysis identified significant enrichment^66–68^ of cytokine binding and signalling, as well as genes involved in HIV Type 1, in the yellow network-module of which HML6_3p21.31c is a member. *Upper X-axes*: − log(10) *p* value of the gene function enrichment analysis. *Y-axes*: Most significant gene function enrichment ontological categories. *Bar-chart titles*: Database used for gene function enrichment analyses.

In the KCL primary motor cortex, HML6_3p21.31c showed significant co-expression in a network enriched for cytokine signalling and binding, viral protein interaction, and responses to EBV and HIV Type 1 infection (see Table 3; Fig. 2). For the genes driving the enrichment for these categories see Supplementary Table S4 and for an extended list of enrichment categories see Supplementary Table S5. This network also showed significant correlation with disease status (Pearson’s r = 0.19, *p* = 0.05) indicating network-wide upregulation in ALS. HML6_3p21.31c was the most significant genomic element to associate with ALS in this network (see Table 3). For genes and ERVs that show differential expression in this network (with an uncorrected *p* value < 0.05) see Supplementary Table S6.

**Table 3:**
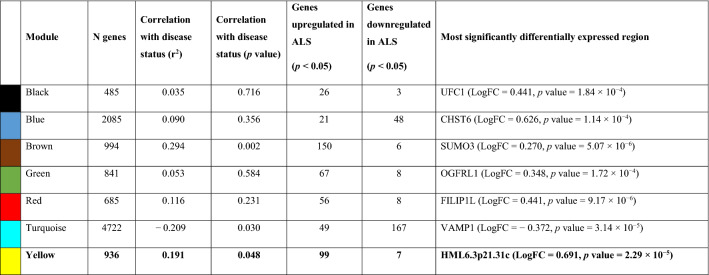
Co-expression analyses using *post-mortem* motor cortex tissue from the KCL cohort.

HML6.3p21.31c co-expresses with genes in the yellow network-module. This network-module positively correlated with ALS disease status, in concordance with increased expression of HML6.3p21.31c in ALS compared to controls. In this network-module, HML6.3p21.31c was the most significantly differentially expressed region (which includes ERVs and genes).

*N Genes:* Number of genes in the module; *Genes up/down regulation in ALS* (*p* < 0.05): Number of genes in the module that show differential expression comparing ALS and controls with a *p* value < 0.05.

For the GSE137810 lateral motor cortex cohort HML6_3p21.31c also showed significant co-expression in a network enriched for cytokine binding and receptor activity, and genes involved in HIV Type 1 infection (see Supplementary Fig. S2; Supplementary Table S7).

The HML6_3p21.31c locus is immediately adjacent to the chemokine receptor cluster on chromosome 3, which has been shown to influence retrovirus HIV viral load and is approximately 50 kb downstream of the HIV coreceptor gene *CCR5*, which is required for HIV entry into the cell. To explore the transcriptional relationship between HML6_3p21.31c and the genes proximal to it we performed Pearson’s correlation analyses using normalised expression estimates in the KCL primary motor cortex dataset. HML6_3p21.31c expression significantly correlated with expression of the HIV coreceptor gene *CCR5* (r = 0.346, *p* value = 0.0002), as well as *CCR1* (r = 0.248, *p* value = 0.009) and Lactoferrin (*LTF*) (r = 0.750, *p* value < 2.2 × 10^−16^), the latter of which have also shown association with HIV. Although HML6_3p21.31.c showed significant co-expression with *CCR5*, *CCR1* and *LTF*, they do not show significant differential expression between ALS and control donors after multiple testing correction (see Supplementary Table S8).

Previous research has demonstrated that ERVs that show significant differential expression in ALS correlate with *TARDBP* expression. We performed expression correlation analyses between a list of 14 genes that associate with ALS and HML6_3p21.31c. A small but significant correlation was identified with *TARDBP* (Pearson’s r = 0.21, *p* value = 0.04) and optineurin (Pearson’s r = − 0.25, *p* value = 0.01). See Supplementary Fig. S3.

### ERVs and genetic risk of ALS

To assess if ERVs that showed increased expression in *post-mortem* ALS increased genetic risk of the disease, we performed ERV-set analysis using MAGMA and three ALS GWAS datasets. For each *post-mortem* RNA-seq dataset analysed, ERVs were divided by their direction of fold-change and filtered by their disease status association with a *p* value ≤ 0.05. ERVs that showed significant increased expression in the KCL primary motor cortex dataset were marginally enriched for SNPs that increased ALS risk (beta = 0.315, S.D. = 0.032, *p* value = 0.039). No other tissue or tissue-source showed enrichment for ALS risk SNPs (see Supplementary Table S9). In addition, we tested whether ERVs belonging to family HML6 show significant enrichment of ALS risk SNPs (beta = 0.199, S.D. = 0.025, *p* value = 0.087).

## Discussion

Using RNA-sequence data from *post-mortem* motor cortex, we found that an individual ERV locus of the HML6 family, HML6_3p21.31c, showed significantly increased expression in ALS. We tested six additional *post-mortem* ALS RNA-sequence datasets and found a significant increase in expression of HML6_3p21.31c in four of them. Increased expression of the ERV locus was found in motor cortex and cerebellum, but not in prefrontal cortex. To understand the potential functional involvement of HML6_3p21.31c in ALS we performed co-expression network analyses. HML6_3p21.31c significantly co-expressed with genes involved in cytokine binding and signalling, interleukin, inflammatory responses, EBV infection, viral protein interaction, HTLV-1 and HIV Type-1 infection.

Previous research has reported differential expression of specific HERV-K HML2 loci on chromosome bands 3q21.2, 7q34, and 10p14 in *post-mortem* ALS, as well as differential expression of HML2 as a family^25,26^. We did not find evidence that these specific loci or the HML2 family were differentially expressed in ALS motor cortex, cerebellum or prefrontal cortex. Our findings concur with recent studies using qPCR in *post-mortem* ALS that also found no differential expression of these loci or the HML2 ERV family^41,42^. The qPCR primers used across all previous studies examining HML2 in ALS would not have amplified HERV-K HML6 loci and therefore could not show association with ALS. Two key differences exist between our study and the studies that originally found differential expression of HML2 loci. We have used methods that are specifically designed to quantify ERV loci-specific transcripts, while accounting for sequence alignment ambiguity and repetitive genomic elements. In addition, the sample-size of our study is larger and has greater power to account for variation across ALS and control populations.

HML6_3p21.31c belongs to the HERV-K(HML6) family of ERVs. HML-6 expression has been found throughout the genome in healthy and diseased tissue^46^. The most recent clinical association was found comparing HIV-1 infected cell cultures with control T cells^47^. In a similar bioinformatic approach to that used in our paper^47^, Grandi et al. found upregulation of HML6_ 19q13.43 in HIV-positive cells, which was the highest expressed ERV of the 3250 tested. HML-6 has also shown association with breast cancer^48^ and melanoma^49^.

HML6_3p21.31c is proximal to and shows significant co-expression with HIV associated genes *CCR5*, *CCR2* and *LTF*. Outside of the HLA locus, *CCR5* is the only gene that associates with HIV susceptibility^50^ and viral load^51^ through genetic inheritance. *CCR5* encodes for a HIV co-receptor^52^, where genetic mutation (*CCR5*-delta 32) reduces receptor functionality and inhibits HIV’s capacity to infect cells^53^. Lactoferrin also exhibits anti-HIV effects^54^, with preliminary evidence of *LTF* polymorphisms influencing maternal transmission of HIV-1 to offspring. Using network analyses, we found genes that co-expressed with HML6_3p21.31c were enriched for genes that associate with HIV Type-1 infection. These included *IL4R*, *CCL3L1*, *CXCR1* and *CCL2*, in addition to genes proximal to the ERV locus (*CCR5*, *CCR2*, *CCR1* and *LTF*).

Comorbidity exists between ALS and HIV and was first identified in 1985^55^. Estimates indicate that 3.5 in 1000 HIV patients develop ALS-like symptoms^56^, and this is now referred to as HIV-associated ALS. Clinical studies have found that HIV-associated ALS symptoms may be reversible through antiretroviral therapies^56^, and there are clinical trials^57^ in ALS patients without HIV infection, currently ongoing. Our results present the first locus-specific link between ALS, ERVs and HIV associated genes. Given that none of our *post-mortem* samples had known HIV-associated ALS, our results support evidence that ERV and HIV-related pathways are important in the wider ALS clinical population.

One limitation of our study is the uncertainty about what is causing upregulation of HML6_3p21.31c in ALS. It may be driven by expression of HIV-associated genes nearby, however HML6_3p21.31c alone showed significant upregulation in this locus, whereas HIV-associated genes did not. HML6_3p21.31c co-expressed with cytokine and HIV-associated genes as part of a wider network. Within this network, HML6_3p21.31c was the most significant genomic element to associate with ALS. Given that ERVs are known to regulate gene expression, our results highlight the potential that HML6_3p21.31c may be key genomic elements in modifying HIV associated and cytokine pathways in ALS.

Enrichment analyses of genes that co-expressed with HML6_3p21.31c in a network included genes associated with infection by several exogenous viruses, including HIV-1, HTLV-1, and EBV, as well as other viral-associated immune response genes. A limitation to our study is that we cannot be certain that this enrichment is not driven by immunological pathways in response to ALS, that parallel immune responses to viruses.

We found HML6.3p21.31c co-expressed with a network that correlated with both neuronal and non-neuronal cell-types. While it cannot be certain that differential expression of HML6.3p21.31c is not influenced by differences in cell composition between ALS and control donors, we integrated surrogate variable analyses into our model which controls for cell heterogeneity across samples^58^. In addition, we found no significant differences in neuron estimates between ALS and controls donors using cell composition analyses.

Anti-retroviral drugs are currently being trialled in ALS^57^. These trials are based upon decades of research showing differential expression of reverse transcriptase activity and ERV transcripts in ALS. While ERV families, and reverse transcriptase activity have shown association with the disease, findings regarding specific ERV transcripts have been less consistent. We found significant upregulation of a specific ERV locus (HML6_3p21.31c) in four *post-mortem* ALS RNA-sequence datasets across three studies. To date, this is the largest analysis of its kind in ALS. HML6_3p21.31c is located at the chemokine receptor gene cluster on chromosome 3 that modifies HIV susceptibility, viral entry into the cell and viral load. Network analyses of HML6_3p21.31c reveal significant correlation with genes involved in cytokine and interleukin activity, and infection with HIV and other exogenous viruses. In summary, our results (1) support the hypothesis that ERVs are involved in ALS pathology, (2) identify a specific ERV locus that shows consistent upregulation in ALS, and (3) identify major pathological pathways in the disease in which ERVs may play a significant role.

## Material and methods

### Samples and cohorts

Samples with *post-mortem* RNA-sequence data were used from three sources: (1) King’s College London (KCL) and the MRC London Neurodegenerative Diseases Brain Bank, (2) Target ALS Human *Post-mortem* Tissue Core and the New York Genome Centre (NYGC) published by Conlon et al.^59^ and Tam et al.^60^ (NCBI GEO ID: GSE137810), and (3) the Florida Mayo Clinic published by Prudencio et al.^61^ (NCBI GEO ID: GSE67196). Hereafter, datasets will be referred to by their respective GEO (GSE) accession number. ALS samples and controls were matched by age and sex where possible for each individual dataset. For a summary of sample frequencies, stratified by project and anatomical location, please see Table 1.

For KCL and the MRC London Neurodegenerative Diseases Brain Bank, frozen human *post-mortem* tissue was taken from primary motor cortex. For GSE137810, frozen human *post-mortem* tissue was taken from motor cortex (medial and lateral), frontal cortex (various locations) and cerebellum. For GSE67196, frozen human *post-mortem* tissue was taken from the lateral hemisphere of the cerebellum, the prefrontal cortex (Brodmann area 9/44), and the primary motor cortex (Brodmann area 4). For KCL and GSE67196^61^, all controls had Braak stage ≤ 2 with the exception of one control in GSE67196^61^.

### RNA-sequencing of *post-mortem* samples

Library preparation and sequencing of RNA are described in https://metronome.nygenome.org/NYGC-RNA-Seq-QC-README_29Jun2017.pdf for GSE137810 data and Prudencio et al.^61^ for the GSE67196 dataset.

For the MRC London Neurodegenerative Diseases Brain Bank please see supplementary methods.

### Experimental design and statistical analyses

#### Differential expression analysis of individual ERVs

All differential expression analyses were performed using DESeq2 controlling disease status, gender, age, *post-mortem* delay, RIN and surrogate variables, where available. All log2 fold-change values reflect changes in expression in comparison to non-ALS controls. All *p* values reported are raw *p* values unless reported otherwise. For multiple testing correction we used independent hypothesis weighting^62^ and Bonferroni corrections, which are reported where applicable.

#### Co-expression and cell-type analyses

To identify which genes significantly co-expressed with ERVs (defined by ERVMap transcript IDs), we performed variance stabilizing transformations (VST) for all required datasets using DESeq2^63^. We corrected normalised expression estimates by covariates sex, age, *post-mortem* delay, RIN and surrogate variables, using jaffelab function cleaningY^64^ to regress them out. Next we used BRETIGEA to estimate cell composition per individual sample using BRETIGEA’s cell marker dataset (50 markers)^45^.

We used weighted correlation network analysis (WGCNA)^65^ alongside VST normalised expression data for the KCL primary motor cortex and GSE137810 lateral motor cortex datasets. WGCNA was performed using a deepsplit of 0 and a minimum module size of 30 genes\ERVs. Next, we analysed if WGCNA modules correlated with disease by performing Pearson’s r correlation between disease status and module eigengene values. We performed the same analysis for cell-type estimates by sample.

To perform co-expression analysis of individual ERVs and genes we used Pearson’s r analyses using PerformanceAnalytics in R (https://github.com/R-Finance/PerformanceAnalytics).

#### Gene function enrichment analyses

Enrichment analyses of gene function was performed using g:profiler and Enrichr, which include KEGG pathways^66–68^. Ontological categories with a term size (number of genes) greater than 1000 are not reported.

#### ERV-set enrichment analysis

To asses if ERVs were enriched for SNPs that modify ALS disease risk we used MAGMA^69^ and three ALS GWAS summary statistics from Nicolas et al.^70^ and van Rheenen and Shatunov et al.^9^. Note that these GWAS have overlapping samples but use alternative statistical models (please see references for more details).

We performed annotation of the 1000 genomes reference build GRCh37/hg19 Phase 3 using a 50 kb flanking window. A 50 kb flanking region was selected as the median length of a haplotype for chromosome 1^71^ and ERV loci regions are small in comparison to protein-coding genes, which diminishes the capacity to identify an enrichment of SNPs that associate with disease. Gene-level analyses were performed on an ad-hoc ERV loci file using ERVMap mapping coordinates lifted over to reference genome GRCh37/hg19 Phase 3. ERV-set analyses were then performed using standard MAGMA protocols.

#### Family-level ERV expression analyses

To test if ERV families were differentially expressed in *post-mortem* ALS we used TETranscripts^72^. TETranscripts uses an expectation maximization algorithm to quantify multi-mapped reads, which is designed to estimate abundance of transcribed transposable elements (like ERVs) that have high sequence fidelity (like the sequence overlap that occurs between ERVs of the same family).

To perform TETranscripts we used RNA-seq bam files derived from all seven datasets tested. ERV families with total read-count less than 10 were discarded. All differential expression analyses of ERV families were performed using DESeq2 controlling disease status, gender, age, *post-mortem* delay, RIN, surrogate variables, where available.

### Ethics declaration

*Post-mortem* tissue samples from King’s College London were collected under the ethical approval of the MRC London Neurodegenerative Diseases Brain Bank and under the regulations of the Human Tissue Act UK 2014. All post-mortem tissue was donated to the MRC London Neurodegenerative Diseases Brain Bank under standard ethical and Human Tissue Act procedures, with informed consent provided by the next of kin. Data generated from this material were anonymized and analysed on a high-performance computing cloud (https://www.maudsleybrc.nihr.ac.uk/facilities/rosalind/) with data protection protocols in accordance with Department of Health Policy (UK) and the security standards set by the National Data Guardian. Ethical approval to process and analyse *post-mortem* samples stored at King’s College London was provided by a local ethics committee at the Institute of Psychiatry, Psychology & Neuroscience, King’s College London, and the MRC London Neurodegenerative Diseases Brain Bank.

## Supplementary Information

Supplementary Information.

## Data Availability

Dataset are available, on reasonable request. The post-mortem genetic and RNA-sequence datasets are available from the corresponding author, the GWAS survival datasets are available through Dr Isabella Fogh, and the Braineac eQTL datasets are available through the UK Brain Expression Consortium.
