# Author Correction: A HML6 endogenous retrovirus on chromosome 3 is upregulated in amyotrophic lateral sclerosis motor cortex

**DOI:** 10.1038/s41598-022-18488-y

**Published:** 2022-08-19

**Authors:** Ashley R. Jones, Alfredo Iacoangeli, Brett N. Adey, Harry Bowles, Aleksey Shatunov, Claire Troakes, Jeremy A. Garson, Adele L. McCormick, Ammar Al‑Chalabi

**Affiliations:** 1 Department of Basic and Clinical Neuroscience, Maurice Wohl Clinical Neuroscience Institute, Institute of Psychiatry, Psychology and Neuroscience, King’s College London, London, SE5 9NU UK; 2 Department of Biostatistics and Health Informatics, Institute of Psychiatry, Psychology and Neuroscience, King’s College London, London, UK; 3 National Institute for Health Research Biomedical Research Centre and Dementia Unit at South London and Maudsley NHS Foundation Trust and King’s College London, London, UK; 4 Social Genetic and Developmental Psychiatry Centre, Institute of Psychiatry, Psychology and Neuroscience, King’s College London, London, UK; 5 NIHR Maudsley Biomedical Research Centre, South London and Maudsley NHS Trust, King’s College London, London, UK; 6 National Institute for Health Research Biomedical Research Centre at Guy’s and St Thomas’ NHS Foundation Trust and King’s College London, London, UK; 7 MRC London Neurodegenerative Diseases Brain Bank, Institute of Psychiatry, Psychology and Neuroscience, King’s College London, London, UK; 8 Division of Infection and Immunity, University College London, London, UK; 9 School of Life Sciences, University of Westminster, London, UK

Correction to: *Scientific Reports* https://doi.org/10.1038/s41598-021-93742-3, published online 12 July 2021

The original version of this Article contained an error in the Funding section.

“This work was funded by the Motor Neurone Disease Association (Grant: Jones/Oct15/958-799) and the ALS Association (Grant: 18-LGCA- 394 "HERV-K molecular studies in ALS"). AAC is an NIHR Senior Investigator. This is an EU Joint Programme - Neurodegenerative Disease Research (JPND) project. The project is supported through the following funding organisations under the aegis of JPND - https://www.jpnd.eu (United Kingdom, Medical Research Council (MR/L501529/1; MR/R024804/1) and Economic and Social Research Council (ES/L008238/1)) and through the Motor Neurone Disease Association. This study represents independent research part funded by the National Institute for Health Research (NIHR) Biomedical Research Centre at South London and Maudsley NHS Foundation Trust and King’s College London.”

now reads:

“This work was funded by the Motor Neurone Disease Association (Grant: Jones/Oct15/958-799), the ALS Association (Grant: 18-LGCA- 394 "HERV-K molecular studies in ALS") and MND Scotland (Grant: "Multi-omics analysis of the role of human endogenous retroviruses in ALS"). AAC is an NIHR Senior Investigator. This is an EU Joint Programme - Neurodegenerative Disease Research (JPND) project. The project is supported through the following funding organisations under the aegis of JPND - https://www.jpnd.eu (United Kingdom, Medical Research Council (MR/L501529/1; MR/R024804/1) and Economic and Social Research Council (ES/L008238/1)) and through the Motor Neurone Disease Association. This study represents independent research part funded by the National Institute for Health Research (NIHR) Biomedical Research Centre at South London and Maudsley NHS Foundation Trust and King’s College London.”

The original Article has been corrected.

