# Supplementary material for: A HML6 endogenous retrovirus on chromosome 3 is upregulated in amyotrophic lateral sclerosis motor cortex
Source: Sci Rep. 2021 Jul 12;11:14283. doi: 10.1038/s41598-021-93742-3 (PMC8275748; doi:10.1038/s41598-021-93742-3)
Supplement: Supplementary file 1 — Supplementary Information. [file 41598_2021_93742_MOESM1_ESM.pdf]

SUPPLEMENTARY INFORMATION

A HML6 endogenous retrovirus on chromosome 3 is upregulated in amyotrophic lateral sclerosis motor cortex

Ashley R Jones[1], Alfredo Iacoangeli[1,2,3], Brett N Adey[2,4], Harry Bowles[1,2,3,5], Aleksey Shatunov[1], Claire Troakes[6], Jeremy A Garson[7], Adele L McCormick[8], Ammar Al-Chalabi[1]

[1]Department of Basic & Clinical Neuroscience, Maurice Wohl Clinical Neuroscience Institute, Institute of Psychiatry, Psychology and Neuroscience, King's College London, London, UK SE5 9NU

[2]Department of Biostatistics and Health Informatics, Institute of Psychiatry, Psychology and Neuroscience, King’s College London, London UK

[3]National Institute for Health Research Biomedical Research Centre and Dementia Unit at South London and Maudsley NHS Foundation Trust and King’s College London, London, UK

[4]Social Genetic and Developmental Psychiatry Centre, Institute of Psychiatry, Psychology & Neuroscience, King's College London, UK; and NIHR Maudsley Biomedical Research Centre, South London and Maudsley NHS Trust, King's College London, UK

[5]National Institute for Health Research Biomedical Research Centre at Guy's and St Thomas' NHS Foundation Trust and King’s College London, London, UK

[6]MRC London Neurodegenerative Diseases Brain Bank, Institute of Psychiatry, Psychology and Neuroscience, King’s College London, London UK

[7]Division of Infection and Immunity, University College London, London, UK

[8]School of Life Sciences, University of Westminster, London, UK

Supplementary Methods

RNA-sequencing protocol for samples from the MRC London Neurodegenerative Diseases Brain Bank (King's College London)

Purification, isolation and quality control

The 100mg frozen tissue blocks were divided; one for RNA purification and the other for DNA. For each sample, a 30mg tissue block for RNA was homogenised using a Qiagen PowerLyzer 24 Homogenizer. Total RNA was purified from the homogenate using the standard protocol of the RNeasy Lipid Tissue Mini Kit (Qiagen), with on-column DNAse digestion. RNA integrity was estimated using Agilent Bioanalyzer 2100's RNA 6000 Nano assays. RNA quantification was performed using a NanoDrop.

Library preparation and RNA sequencing

Library preparation was performed using the standard Illumina TruSeq Stranded Total RNA Sample Preparation Guide with Ribo-Zero Human/Mouse/Rat (October 2013 Rev E.). Fragmentation steps were tailored to degrees of degraded RNA samples using Agilent Bioanalyzer 2100's Nano assay results from the previous section. Libraries were validated using an Agilent Bioanalyzer 2100 to assess fragment size distribution. Library concentrations were estimated using a Qubit RNA High Sensitivity Assay Kit. Nanomolar (nM) concentrations were estimated using: nM = ng/ul X (1500/Average bp). Libraries were sequenced using Illumina HiSeq 4000 flow cells with 150bp paired-end reads with a target depth of 30 million clusters (60 million reads per sample).

Post-sequencing Quality Control, Alignment and Normalisation

For all RNA-sequence datasets we used MultiQC[1] to assess read quality pre- and post- alignment. SortMeRNA[2] was used to filter and remove ribosomal RNA transcripts using rRNA reference databases. Clip adapter sequences and low-quality reads were removed using BBDuk (http://jgi.doe.gov/data-and-tools/bb-tools/). We used STAR v2.7[3] and GRCh38.89 reference assemblies for alignment of RNA-sequenced reads.

To quantify locus-specific expression of ERVs we used a modified ERVMap pipeline and the ERVMap database. ERVMap ERV loci are selected by their potential to be autonomous full-length proviruses and provides a reference ERVome for accurate locus-specific ERV quantification using RNA-seq data (3237 ERV loci). Please see Tokuyama et al. 2018[4] for details on the ERV-specific RNA-sequencing pipeline. We modified the pipeline, replacing TopHat[5] aligner with STAR v2.7[3]. ERVMap transcript identifiers were matched with RepeatMasker loci identifiers[6].

Tximport[7] and DESeq2[8] was used to import transcript abundances into R Statistics 4.0.1. Read counts with a total less than 10 were removed. Where possible, the following sample-level phenotype and batch data was imported into R Statistics: disease status, gender, age (categorised into quintiles), *post-mortem* delay (categorised into quintiles), RIN (RNA Integrity Number), and flow-cell. Surrogate variable analyses were performed using SVA and SVAseq[9], while controlling for known covariates, where surrogate variables were appended to each sample. This is to estimate heterogeneity due to extraneous variables (such as cell heterogeneity and missing covariates). Data was normalised using variance stabilising transformations (VST) for use with analyses other than DESeq2 differential expression analyses.

1. Ewels, P., Magnusson, M., Lundin, S. & Käller, M. MultiQC: summarize analysis results for multiple tools and samples in a single report. *Bioinformatics* **32**, 3047–3048 (2016).
2. Kopylova, E., Noé, L. & Touzet, H. SortMeRNA: fast and accurate filtering of ribosomal RNAs in metatranscriptomic data. *Bioinformatics* **28**, 3211–3217 (2012).
3. Dobin, A. *et al.* STAR: ultrafast universal RNA-seq aligner. *Bioinformatics* **29**, 15–21 (2013).
4. Tokuyama, M. *et al.* ERVmap analysis reveals genome-wide transcription of human endogenous retroviruses. *Proc. Natl. Acad. Sci. U. S. A.* **115**, 12565–12572 (2018).
5. Trapnell, C., Pachter, L. & Salzberg, S. L. TopHat: discovering splice junctions with RNA-Seq. *Bioinformatics* **25**, 1105–1111 (2009).
6. Smit, A., Hubley, R. & Green, P. RepeatMasker.
7. Soneson, C., Love, M. I. & Robinson, M. D. Differential analyses for RNA-seq: transcript-level estimates improve gene-level inferences. *F1000Research* **4**, 1521 (2016).
8. Love, M. I., Huber, W. & Anders, S. Moderated estimation of fold change and dispersion for RNA-seq data with DESeq2. *Genome Biol.* **15**, 550 (2014).
9. Leek, J. T. svaseq: removing batch effects and other unwanted noise from sequencing data. *Nucleic Acids Res.* **42**, e161–e161 (2014).
10. Kanehisa, M., Furumichi, M., Sato, Y., Ishiguro-Watanabe, M. & Tanabe, M. KEGG: Integrating viruses and cellular organisms. *Nucleic Acids Res.* **49**, D545–D551 (2021).
11. Kanehisa, M. Toward understanding the origin and evolution of cellular organisms. *Protein Science* vol. 28 1947–1951 (2019).
12. Kanehisa, M. & Goto, S. KEGG: Kyoto Encyclopedia of Genes and Genomes. *Nucleic Acids Research* vol. 28 27–30 (2000).

Supplementary Figures

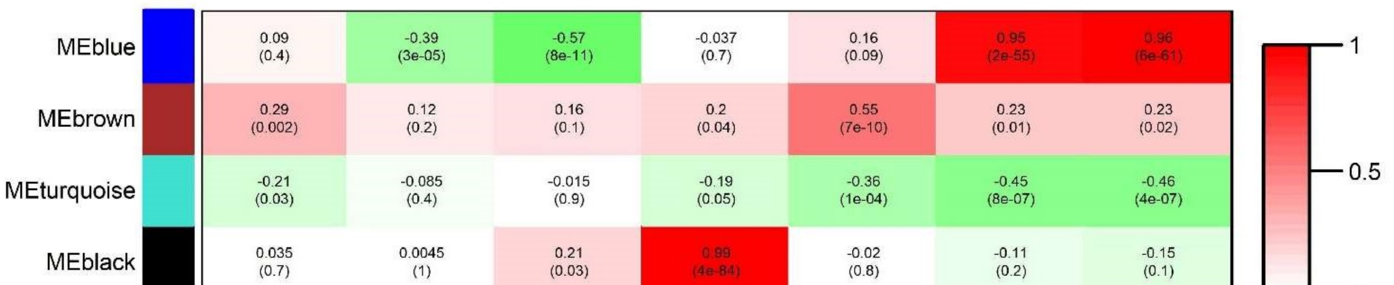

Figure S1. Co-expression analyses using *post-mortem* ALS motor cortex tissue. HML6.3p21.31c co-expresses with genes in the yellow network-module. This network-module significantly positively correlated with ALS disease status, in concordance with increased expression of HML6.3p21.31c in ALS compared to controls. The yellow network-module co-expresses with gene markers for multiple cell types and it is unlikely that this network is cell-type specific.

Co-expression network modules. Each network-module represents genes that co-express in *post-mortem* ALS motor cortex. *Y-Axis*: Network-module allocated colour. *X-axis*: Network-wide expression (eigen-network value) correlated with sample disease status and estimated brain cell count. *Box colours*: Red corresponds towards positive correlation, whereas green corresponds towards negative correlation, of network-modules with variable (on the *Y-Axis*). *Module values (within boxes)*: Pearson's r correlation value. P-value from Pearson's R analysis is in paratheses.

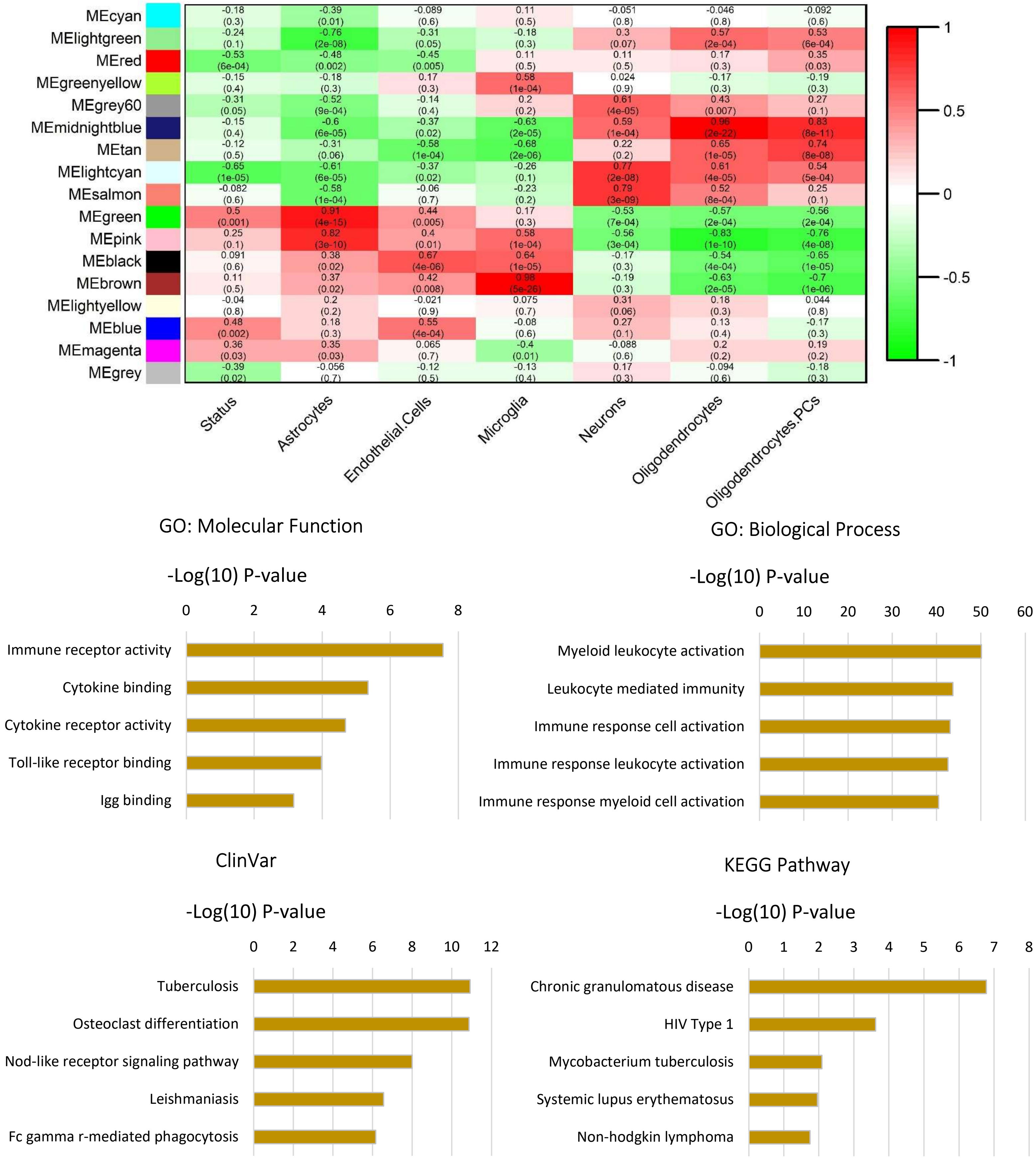
MEcyan
MElightgreen
MEred
MEgreenyellow
MEgrey60
MEmidnightblue
MEtan
MElightcyan
MEsalmon
MEgreen
MEpink
MEblack
MEbrown
MElightyellow
MEblue
MEmagenta
MEgrey
-0.18 (0.3) -0.39 (0.01) -0.089 (0.6) 0.11 (0.5) -0.051 (0.8) -0.046 (0.8) -0.092 (0.6)
-0.24 (0.1) -0.76 (2e-08) -0.31 (0.05) -0.18 (0.3) 0.3 (0.07) 0.57 (2e-04) 0.53 (6e-04)
-0.53 (6e-04) -0.48 (0.002) -0.45 (0.005) 0.11 (0.5) 0.11 (0.5) 0.17 (0.3) 0.35 (0.03)
-0.15 (0.4) -0.18 (0.3) 0.17 (0.3) 0.58 (1e-04) 0.024 (0.9) -0.17 (0.3) -0.19 (0.3)
-0.31 (0.05) -0.52 (9e-04) -0.14 (0.4) 0.2 (0.2) 0.61 (4e-05) 0.43 (0.007) 0.27 (0.1)
-0.15 (0.4) -0.6 (6e-05) -0.37 (0.02) -0.63 (2e-05) 0.59 (1e-04) 0.96 (2e-22) 0.83 (8e-11)
-0.12 (0.5) -0.31 (0.06) -0.58 (1e-04) -0.68 (2e-06) 0.22 (0.2) 0.65 (1e-05) 0.74 (8e-08)
-0.65 (1e-05) -0.61 (6e-05) -0.37 (0.02) -0.26 (0.1) 0.77 (2e-08) 0.61 (4e-05) 0.54 (5e-04)
-0.082 (0.6) -0.58 (1e-04) -0.06 (0.7) -0.23 (0.2) 0.79 (3e-09) 0.52 (8e-04) 0.25 (0.1)
0.5 (0.001) 0.91 (4e-15) 0.44 (0.005) 0.17 (0.3) -0.53 (7e-04) -0.57 (2e-04) -0.56 (2e-04)
0.25 (0.1) 0.82 (3e-10) 0.4 (0.01) 0.58 (1e-04) -0.56 (3e-04) -0.83 (1e-10) -0.76 (4e-08)
0.091 (0.6) 0.38 (0.02) 0.67 (4e-06) 0.64 (1e-05) -0.17 (0.3) -0.54 (4e-04) -0.65 (1e-05)
0.11 (0.5) 0.37 (0.02) 0.42 (0.008) 0.98 (5e-26) -0.19 (0.3) -0.63 (2e-05) -0.7 (1e-06)
-0.04 (0.8) 0.2 (0.2) -0.021 (0.9) 0.075 (0.7) 0.31 (0.06) 0.18 (0.3) 0.044 (0.8)
0.48 (0.002) 0.18 (0.3) 0.55 (4e-04) -0.08 (0.6) 0.27 (0.1) 0.13 (0.4) -0.17 (0.3)
0.36 (0.03) 0.35 (0.03) 0.065 (0.7) -0.4 (0.01) -0.088 (0.6) 0.2 (0.2) 0.19 (0.2)
-0.39 (0.02) -0.056 (0.7) -0.12 (0.5) -0.13 (0.4) 0.17 (0.3) -0.094 (0.6) -0.18 (0.3)
1
0.5
0
-0.5
-1
Status
Astrocytes
Endothelial.Cells
Microglia
Neurons
Oligodendrocytes
Oligodendrocytes.PCs
GO: Molecular Function
-Log(10) P-value
0 2 4 6 8
Immune receptor activity
Cytokine binding
Cytokine receptor activity
Toll-like receptor binding
Igg binding
GO: Biological Process
-Log(10) P-value
0 10 20 30 40 50 60
Myeloid leukocyte activation
Leukocyte mediated immunity
Immune response cell activation
Immune response leukocyte activation
Immune response myeloid cell activation
ClinVar
-Log(10) P-value
0 2 4 6 8 10 12
Tuberculosis
Osteoclast differentiation
Nod-like receptor signaling pathway
Leishmaniasis
Fc gamma r-mediated phagocytosis
KEGG Pathway
-Log(10) P-value
0 1 2 3 4 5 6 7 8
Chronic granulomatous disease
HIV Type 1
Mycobacterium tuberculosis
Systemic lupus erythematosus
Non-hodgkin lymphoma

Figure S2. Co-expression analyses using *post-mortem* ALS lateral motor cortex tissue. HML6.3p21.31c co-expresses with genes in the brown network-module.

Top. Co-expression network modules. Each network-module represents genes that co-express in *post-mortem* ALS motor cortex. *Y-Axis*: Network-module allocated colour. *X-axis*: Network-wide expression (eigen-network value) correlated with sample disease status and estimated brain cell count. *Box colours*: Red corresponds towards positive correlation, whereas green corresponds towards negative correlation, of network-modules with variable (on the *Y-Axis*). *Module values (within boxes)*: Pearson's r correlation value. P-value from Pearson's R analysis is in paratheses.

Bottom. Gene function enrichment analyses of brown network-module. This analysis identified significant enrichment of cytokine binding, as well as genes involved in HIV Type 1, similar to yellow module-network identified using *post-mortem* motor cortex from the GSE137810 lateral motor cortex dataset. *Upper X-axes*: -log(10) P-value of the gene function enrichment analysis. Y-axes: Most significant gene function enrichment ontological categories. Bar-chart titles: Database used for gene function enrichment analyses.

GENE SYMBOL	PEARSON'S R	P-VALUE
**TARDBP**	0.210	0.041
**FUS**	0.172	0.092
**UBQLN2**	0.170	0.095
**GRN**	0.150	0.140
**SCFD1**	0.031	0.755
**PFN1**	0.002	0.986
**C9ORF72**	-0.006	0.950
**TBK1**	-0.034	0.739
**NEK1**	-0.056	0.579
**ATXN2**	-0.077	0.445
**HNRNPA1**	-0.098	0.333
**SOD1**	-0.117	0.249
**UNC13A**	-0.196	0.056
**OPTN**	-0.253	0.014

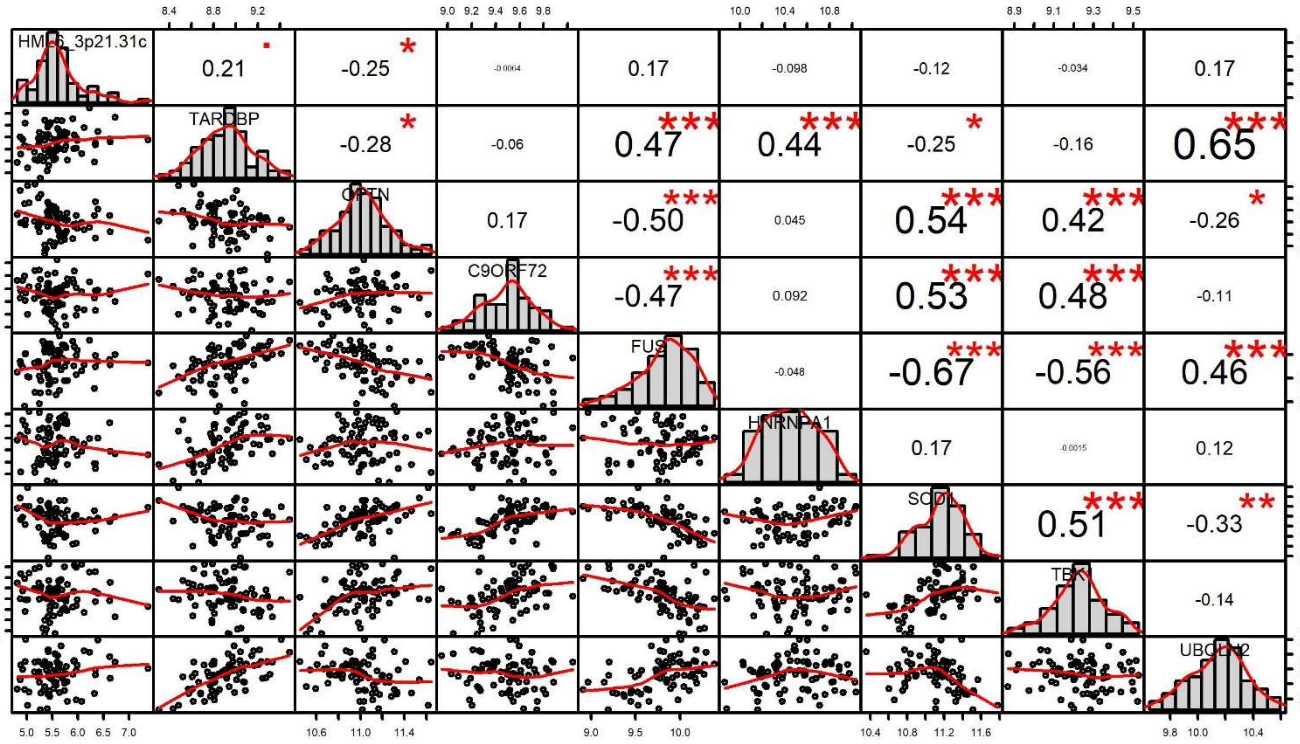

Supplementary Fig S3. Pearson's r correlation of HML6_3p21.31c expression with 14 ALS genes in ALS motor cortex. HML6_3p21.31c correlates with TARDBP, where r = 0.210 and p-value = 0.041. **Left**: Table showing Pearson's r correlation of HML6_3p21.31c expression with 14 ALS genes with corresponding p-value. **Right.** Matrix of expression correlations. *Lower-left: S*catterplots of expression values across samples for ERVs and\or genes represented on X and Y axes. *Centre-Diagonal*: The distribution of HML6_3p21.31c and ALS gene expression values over samples. *Upper-right:* Pearson's r correlation value of expression between genes, and gene and HML6_3p21.31c, with statistical significance values represented as star. * $p < 0.05$, ** $p < 0.01$, *** $p < 0.001$.

Supplementary Tables

					Motor Cortex			*Cerebellum*		*Frontal Cortex*	
Locus ID	**Previous designations**	**Chr.**	**Start**	**End**	**Log2 FC Primary KCL**	**Log2 FC Lateral GSE137810**	**Log2 FC Medial GSE137810**	**Log2 FC Cerebellum GSE137810**	**Log2 FC Cerebellum GSE67196**	**Log2 FC FCx GSE137810**	**Log2 FC PFC GSE67196**
HML2_3q12.2	HERV-K(I); chr3q21.2_K-4	3	125890457	125899596	-0.05 (0.54)	0.20 (0.15)	0.20 (0.16)	-0.06 (0.75)	0.91 (0.05)	0.05 (0.70)	0.28 (0.41)
HML2_7q34	c7_C; chr7q34_K-15	7	141751124	141756138	-0.05 (0.52)	-0.04 (0.61)	-0.20 (0.11)	0.21 (0.07)	0.10 (0.68)	0.10 (0.28)	0.08 (0.75)
HML2_8p23.1	HERV-k115	8	7497023	7507805	-0.24 (0.15)	-0.33 (0.15)	0.35 (0.31)	-0.03 (0.92)	-0.36 (0.54)	-0.35 (0.11)	0.05 (0.89)
HML2_10p14	c10_A; chr10p14_K-16	10	6824177	6833641	0.06 (0.68)	0.03 (0.89)	0.22 (0.32)	0.23 (0.35)	-0.51 (0.35)	-0.01 (0.96)	-0.42 (0.23)
HML2_19p12	HERV-k113	19	21880627	21890401	-0.26 (0.12)	-0.30 (0.20)	-0.14 (0.65)	0.11 (0.69)	0.95 (0.11)	-0.10 (0.65)	0.57 (0.18)

Supplementary Table S1. Differential expression analysis of ERV loci previously studied in ALS. Values for Motor Cortex, Cerebellum, and Frontal Cortex are -log2 Fold-Change with p-value with parentheses from the differential expression analyses. Previous designations: Refers to previous loci IDs used in Li et al. 2015 and Mayer et al. 2018. Chr.: Chromosome, FC: Fold-Change, KCL: King's College London, FCx: Frontal Cortex, PFC: Prefrontal cortex

ERVMap ID	Family	Chr	Base Position Start	Base Position End	Log2 FC Primary KCL	Log2 FC Lateral GSE137810	Log2 FC Medial GSE137810	P-value Primary KCL	P-value Lateral GSE137810	P-value Medial GSE137810	Stouffer's Meta-analysis Z	Stouffer's Meta-analysis P
943	HML6	3	46426676	46433564	0.691	0.642	0.645	2.29E-05	0.004	0.0479	5.413	6.18E-08
849	HERVE	3	848018	853278	-0.436	-0.311	-0.74	0.001	0.153	0.0043	-4.335	1.46E-05
1351	HERV9	4	27974870	27981380	-0.295	-0.835	-0.967	0.079	2.89E-04	0.0025	-3.915	9.05E-05
3682	HERVL	12	5820733	5826818	0.343	0.427	0.75	0.014	0.054	0.0027	3.893	9.89E-05
654	MER50	2	115116051	115124440	0.102	0.235	0.74	0.221	0.052	3.72E-06	3.417	6.33E-04
1782	Unclassifiable	5	24296854	24314450	0.424	0.393	0.342	0.009	0.074	0.2854	3.276	1.05E-03
4758	HML6	19	51976954	51992228	0.154	0.297	0.104	0.037	0.003	0.3927	3.194	0.001
2251	HERV9	6	86671994	86681327	0.188	0.354	1.023	0.201	0.076	9.77E-05	3.139	0.002
4438	HML3	17	31631005	31637132	0.195	0.689	0.981	0.241	0.004	0.0032	3.127	0.002
2731	HERVE	8	11931841	11936717	-0.382	-0.126	-0.541	0.009	0.579	0.0578	-3.113	0.002
3409	HUERSP3	11	17370179	17379293	-0.198	-0.157	-0.063	0.010	0.073	0.5320	-3.092	0.002
4491	MER41	18	405895	418808	0.232	0.562	0.404	0.068	0.011	0.1490	3.008	0.003
3078	HERV9	9	78431299	78443032	0.396	-0.228	0.367	0.001	0.281	0.1106	2.989	0.003
2839	HML1	8	60493460	60499925	0.257	0.298	0.317	0.031	0.094	0.1831	2.940	0.003
500	MER41	2	33051995	33055372	-0.173	-0.566	-0.822	0.277	0.015	0.0023	-2.916	0.004
2636	HERVT	7	134548503	134558366	0.303	0.275	0.68	0.052	0.233	0.0240	2.914	0.004
6171	HML2	1	207632284	207641252	0.241	0.281	1.055	0.144	0.233	0.0012	2.853	0.004
967	HERVH	3	58585013	58595736	0.345	0.257	0.184	0.018	0.178	0.3894	2.832	0.005
2077	HERVW	6	13881106	13887724	0.279	0.813	0.095	0.091	5.69E-04	0.7775	2.816	0.005
W-11	NA	2	164657909	164659611	0.197	0.292	0.811	0.177	0.128	0.0027	2.791	0.005
3533	HERV3	11	68867422	68874590	-0.193	-0.243	-0.396	0.097	0.090	0.0380	-2.786	0.005
3436	HUERSP3	11	34897253	34907021	0.176	0.353	0.482	0.146	0.058	0.0213	2.764	0.006
W-27	NA	4	138621785	138627199	0.299	0.42	0.37	0.060	0.056	0.2084	2.763	0.006
ERVV-1	NA	19	53014330	53016580	-0.422	-0.028	-0.514	0.012	0.905	0.1245	-2.755	0.006
1439	MER41	4	67450492	67463204	0.144	0.263	0.17	0.078	0.037	0.1920	2.745	0.006
944	HERVS	3	46417555	46423902	0.273	0.779	0.102	0.101	9.16E-04	0.7506	2.736	0.006
3825	HERVL	12	64130613	64136206	0.284	0.143	0.24	0.021	0.421	0.2158	2.718	0.007
1412	HERV4	4	55938234	55948225	0.387	0.065	0.547	0.022	0.780	0.0942	2.673	0.008
4615	HERVIP	19	9709655	9722342	0.222	0.259	0.745	0.160	0.193	0.0064	2.657	0.008
4790	Unclassifiable	19	57487176	57500813	-0.085	-0.177	-0.21	0.134	0.056	0.0676	-2.639	0.008
4818	HARLEQUIN	20	15980069	15986191	0.332	0.476	-0.011	0.036	0.021	0.9710	2.634	0.008

6238	HML5	1	242212992	242217888	-0.101	-0.483	-0.517	0.318	0.011	0.0178	-2.633	0.008
1908	HERVH	5	96164809	96172177	0.175	0.329	0.57	0.232	0.052	0.0132	2.625	0.009
5346	HERVH	X	37451927	37461065	0.193	0.056	0.51	0.071	0.723	0.0100	2.599	0.009

Supplementary Table S2a. Differential expression analysis of transcripts in **motor cortex**, with Log2 Fold-Change, P-value from differential expression analyses and Stouffer's meta-analysis. Only transcripts with Stouffer's method p-value < 0.01. For this table, ERVMap identifiers are used. Chr: Chromosome, KCL: King's College London, MC: Motor Cortex, FC: Fold-change

ERVMap ID	Family	Chr	Base Position Start	Base Position End	Log2 FC Cerebellum GSE137810	Log2 FC Cerebellum GSE67196	P-value Cerebellum GSE137810	P-value Cerebellum GSE67196	Stouffer's Meta-analysis Z	Stouffer's Meta-analysis P
4213	HERVH	14	92041754	92049863	0.657	0.618	2.87E-08	0.076	5.824	5.76E-09
2152	HML5	6	57069286	57080996	0.545	1.031	1.43E-06	1.22E-04	5.813	6.13E-09
5909	HERVH	1	65637767	65648036	0.926	0.624	3.54E-07	0.054	5.443	5.23E-08
921	HERVH	3	37205387	37210420	0.866	1.002	2.05E-06	0.025	5.221	1.78E-07
4180	LTR19	14	70536592	70544307	0.558	0.784	2.08E-06	0.045	5.141	2.73E-07
2519	HERVFC	7	64834894	64840158	0.431	0.874	1.58E-04	0.010	4.409	1.04E-05
4763	HML6	19	52408080	52414733	0.313	0.781	2.32E-04	0.006	4.379	1.19E-05
4279	HERVW	15	55304849	55312434	0.57	0.543	5.42E-05	0.103	4.347	1.38E-05
4617	HML6	19	11853296	11860978	0.385	0.898	2.09E-04	0.012	4.322	1.55E-05
6228	HERVIP	1	235670260	235675550	0.4	1.284	2.23E-04	0.012	4.309	1.64E-05
1439	MER41	4	67450492	67463204	0.433	0.847	1.59E-04	0.024	4.308	1.65E-05
4678	HERVH	19	23340354	23353938	0.266	1.076	9.19E-04	4.60E-04	4.279	1.88E-05
1080	LTR19	3	119978069	119990309	0.418	0.027	8.95E-06	0.960	4.212	2.53E-05
536	HERVW	2	53755318	53760155	0.393	0.81	1.00E-04	0.110	4.198	2.70E-05
ERVW-26	NA	14	45019484	45023694	0.412	1.273	2.68E-04	0.030	4.156	3.23E-05
2518	HERVH	7	64679994	64686561	0.553	1.419	4.42E-04	0.015	4.118	3.83E-05
1250	HERVW	3	179055106	179062330	0.627	0.498	4.89E-05	0.422	4.100	4.13E-05
2829	MER41	8	54070287	54074793	0.46	0.459	1.03E-04	0.379	3.957	7.60E-05
5429	LTR46	X	63426517	63434693	0.381	0.55	9.59E-04	0.026	3.851	1.17E-04
4830	HERVE	20	24919441	24933027	-0.968	0.065	4.31E-05	0.922	-3.833	1.27E-04
6062	HML2	1	150628158	150635776	0.383	0.143	1.28E-04	0.539	3.820	1.33E-04
ERV3-1	NA	7	64990354	65006746	0.387	0.449	3.70E-04	0.185	3.799	1.45E-04
4533	HERVH	18	31803769	31810043	1.022	0.54	1.91E-04	0.437	3.780	1.57E-04
ERVW-17	NA	6	106228135	106235814	0.38	1.752	0.002	0.007	3.770	1.63E-04
4259	HERVH	15	42884919	42890353	0.489	1.167	8.99E-04	0.053	3.770	1.63E-04
4309	HML3	15	89232354	89239939	-0.94	0.626	1.46E-05	0.305	-3.759	1.70E-04
1073	HERVW	3	115742107	115757987	-0.974	-0.718	3.51E-04	0.297	-3.719	2.00E-04

1712	HARLEQUIN	4	173098722	173106034	0.617	2.192	0.005	0.002	3.714	2.04E-04
3866	HML5	12	88173234	88183818	0.326	0.909	0.002	0.032	3.698	2.17E-04
4361	HERVFB	16	30541068	30546762	0.721	0.834	0.001	0.073	3.637	2.76E-04
2653	HERVH	7	141575646	141584446	0.557	1.02	0.001	0.079	3.589	3.32E-04
3367	HERV9	10	119883734	119890745	0.409	2.224	0.016	1.29E-04	3.525	4.23E-04
750	HUERSP2	2	174202251	174216415	0.302	1.117	0.004	0.019	3.484	4.94E-04
2095	HERVW	6	24667710	24680423	0.401	0.594	0.002	0.157	3.418	6.32E-04
3943	HERVH	13	36316324	36321264	0.421	1.11	0.004	0.030	3.399	6.77E-04
4849	HERVIP	20	49281127	49287343	0.349	0.756	0.004	0.039	3.374	7.41E-04
5918	HERVH	1	70366861	70372373	0.572	0.07	5.05E-04	0.836	3.354	7.96E-04
1281	HERVIP	3	197242999	197248821	0.449	0.622	0.001	0.306	3.352	8.01E-04
5477	HML5	X	73655974	73666201	0.319	1.536	0.010	0.006	3.338	8.45E-04
2776	HERVE	8	41590932	41598326	0.305	0.769	0.005	0.039	3.336	8.49E-04
4097	HERVIP	14	30965848	30972548	0.356	0.787	0.003	0.114	3.328	8.74E-04
2673	HERVH	7	150307239	150313286	0.806	0.902	0.002	0.191	3.324	8.88E-04

Supplementary Table S2b. Differential expression analysis of transcripts in **cerebellum**, with Log2 Fold-Change, P-value from differential expression analyses and Stouffer's meta-analysis. Only transcripts with Stouffer's method p-value < 0.0001. For this table, ERVMap identifiers are used. Chr: Chromosome, FC: Fold-Change

ERVMap ID	Family	Chr	Base Position Start	Base Position End	Log2 FC FCx GSE137810	Log2 FC PFC GSE67196	P-value FCx GSE137810	P-value PFC GSE67196	Stouffer's Meta-analysis Z	Stouffer's Meta-analysis P
4410	HERVIP	16	72064268	72073376	-0.871	0.147	4.70E-06	0.683	-4.137	3.52E-05
2716	HERVE	8	8033824	8046265	-0.91	0.345	3.21E-06	0.369	-4.037	5.41E-05
5872	HERVH	1	45316433	45321282	-0.669	-0.05	6.00E-05	0.903	-3.796	1.47E-04
4258	HERVIP	15	41587553	41592735	-0.79	0.099	1.29E-04	0.727	-3.457	5.45E-04
6262	HERVH	22	16611311	16616782	0.501	0.584	0.003	0.061	3.441	5.79E-04
3301	HML1	10	80104700	80116174	-0.734	-0.408	0.001	0.299	-3.409	6.52E-04
3245	HERVH	10	45571381	45577314	0.477	0.355	9.92E-04	0.382	3.389	7.02E-04
2766	HERV1	8	38081998	38091940	-0.498	0.025	3.63E-04	0.952	-3.314	9.21E-04
3433	HERVFB	11	31608174	31609536	0.734	0.027	7.13E-04	0.908	3.206	0.001
744	HERVH	2	169129439	169137549	0.701	0.293	0.002	0.483	3.199	0.001
5814	HERVFB	1	6560513	6570438	-0.734	-0.021	8.10E-04	0.952	-3.154	0.002
4666	HARLEQUIN	19	22355407	22362466	-0.723	0.069	5.85E-04	0.831	-3.141	0.002
4271	HERV9	15	51357586	51367580	0.495	0.556	0.005	0.160	3.138	0.002
4215	HERVIP	14	92622416	92629608	-0.631	0.053	6.45E-04	0.874	-3.135	0.002
5840	HERVE	1	20153945	20160366	-0.787	0.188	3.48E-04	0.533	-3.124	0.002
4761	HML6	19	52472460	52485485	-0.439	-0.447	0.003	0.283	-3.123	0.002
4862	HERV1	21	14115153	14124623	-0.597	-0.429	0.003	0.302	-3.109	0.002
3586	HERVH	11	89381303	89387559	0.7	-0.044	7.33E-04	0.874	3.102	0.002
559	HERVE	2	64886311	64899831	-0.692	0.066	7.28E-04	0.867	-3.101	0.002
2984	HML4	8	145055668	145066696	-0.236	-0.392	0.024	0.006	-3.081	0.002
1412	HERV4	4	55938234	55948225	0.615	0.581	0.006	0.163	3.048	0.002
1104	HML3	3	126093176	126102115	-0.56	-0.094	0.002	0.808	-2.966	0.003
2636	HERVT	7	134548503	134558366	-0.556	-0.626	0.009	0.131	-2.962	0.003
6094	HERV9	1	169661627	169670891	-0.68	-0.021	0.002	0.928	-2.952	0.003
K-64	NA	X	154608421	154615762	-0.685	0.006	0.002	0.978	-2.932	0.003
2621	HERVH	7	124957310	124967789	0.454	0.38	0.006	0.284	2.931	0.003
4511	MER57	18	13700698	13707821	0.399	0.146	0.004	0.666	2.860	0.004

2946	HERVH	8	119873658	119878636	0.371	0.523	0.017	0.080	2.859	0.004
5657	HARLEQUIN	X	115537907	115545511	0.46	0.404	0.011	0.243	2.790	0.005
1303	HML2	4	9121944	9131359	-0.396	-0.172	0.006	0.604	-2.775	0.006
4523	HERVH	18	28208151	28214533	-0.617	0.148	0.002	0.592	-2.774	0.006
1302	HERVE	4	9094449	9108450	-0.509	0.418	6.96E-04	0.253	-2.767	0.006
3513	HERVE	11	61093577	61099813	-0.698	0.105	0.002	0.650	-2.758	0.006
2090	HERVL	6	19399478	19406918	-0.616	-0.001	0.003	0.997	-2.753	0.006
3395	MER66	11	6747291	6754907	0.313	0.789	0.039	0.021	2.743	0.006
4613	HERVH	19	9741761	9751748	0.251	0.073	0.006	0.606	2.737	0.006
4189	HERVL	14	80224809	80231256	-0.575	0.024	0.003	0.918	-2.729	0.006
4894	HERVIP	21	36104267	36109979	0.256	1.074	0.153	9.04E-05	2.722	0.006
4361	HERVFB	16	30541068	30546762	0.344	1.115	0.062	0.006	2.708	0.007
4804	HML6	20	1396937	1407244	-0.682	0.151	0.002	0.667	-2.681	0.007
4506	HERVL	18	11130174	11134262	0.602	-0.003	0.005	0.989	2.645	0.008
2727	HML5	8	9799846	9814148	-0.581	-0.146	0.010	0.527	-2.634	0.008
W-132	NA	14	58122279	58126144	0.327	0.601	0.024	0.146	2.627	0.009
686	HERVIP	2	132139786	132147993	0.443	0.388	0.017	0.262	2.624	0.009
3226	HERVT	10	37937011	37944189	-0.425	0.365	0.002	0.383	-2.596	0.009

Supplementary Table S2c. Differential expression analysis of transcripts in **frontal cortex**, with Log2 Fold-Change, P-value from differential expression analyses and Stouffer's meta-analysis. Only transcripts with Stouffer's method p-value < 0.0001. For this table, ERVMap identifiers are used. Chr: Chromosome, FCx: Frontal Cortex, PFC: Prefrontal Cortex, FC: Fold-change

ERV Family	Log2 FC Primary KCL	Log2 FC Lateral GSE137810	Log2 FC Medial GSE137810	P-value Primary KCL	P-value Lateral GSE137810	P-value Medial GSE137810	Stouffer's Meta-analysis Z	Stouffer's Meta-analysis P	MAGMA Beta	MAGMA P-value
**HERV3**	0.116	0.103	0.028	0.017	0.250	0.837	2.547	0.011	-0.177	0.897
**HERVE**	-0.090	-0.024	-0.121	0.042	0.816	0.469	-1.812	0.070	-0.062	0.749
**HML5**	0.073	-0.005	-0.037	0.072	0.960	0.771	1.469	0.142	0.065	0.274
**HERVFRD**	-0.188	0.126	-0.165	0.080	0.194	0.158	-1.424	0.154	0.510	0.041
**HERV9**	0.033	0.130	-0.074	0.228	0.165	0.570	1.167	0.243	0.073	0.142
**MER61**	0.737	0.169	0.041	0.515	0.064	0.631	1.072	0.284	1.077	0.014
**HML4**	-0.007	-0.087	-0.111	0.931	0.536	0.391	-1.059	0.289	-0.324	0.852
**HML2**	0.060	-0.001	-0.198	0.190	0.996	0.155	-0.978	0.328	-0.193	0.907
**HERVL**	0.042	0.108	-0.083	0.194	0.256	0.547	0.896	0.370	0.000	0.499
**PABLB**	-0.150	0.139	-0.048	0.028	0.220	0.744	-0.833	0.405	-0.021	0.525
**HML1**	-0.081	0.066	-0.113	0.395	0.689	0.465	-0.814	0.416	0.120	0.216
**HERVW**	0.014	0.051	-0.133	0.617	0.586	0.272	-0.777	0.437	0.073	0.267
**HERVI**	0.055	0.039	-0.123	0.540	0.719	0.330	-0.509	0.611	-0.062	0.592
**HERVH**	-0.109	0.089	0.124	0.064	0.423	0.422	-0.168	0.867	-0.052	0.911
**HERVH48**	-0.051	0.039	0.075	0.376	0.725	0.601	0.080	0.937	0.196	0.186
**HML6**	0.075	0.033	-0.120	0.153	0.712	0.286	-0.064	0.949	0.199	0.087
**HML3**	-0.002	0.054	-0.069	0.960	0.584	0.618	-0.057	0.955	0.113	0.072

Supplementary Table S3a. Differential expression analysis of ERV families using TETranscripts in **motor cortex**, with Log2 Fold-Change, P-value from differential expression analyses, Stouffer's meta-analysis and MAGMA ERV-set analysis using ALS GWAS. Chr: Chromosome, KCL: King's College London, FC: Fold-change

ERV Family	Log2 FC Cerebellum GSE137810	Log2 FC Cerebellum GSE67196	P-value Cerebellum GSE137810	P-value Cerebellum GSE67196	Stouffer's Meta-analysis Z	Stouffer's Meta-analysis P	MAGMA Beta	MAGMA P-value
**HERVH48**	0.309	0.593	0.009	0.017	3.326	0.001	0.196	0.186
**HML5**	0.061	0.584	0.569	0.002	3.126	0.002	0.065	0.274
**PABLB**	0.038	0.613	0.719	0.011	2.570	0.010	-0.021	0.525
**HERVI**	0.138	0.461	0.158	0.024	2.568	0.010	-0.062	0.592
**HERVW**	0.074	0.505	0.500	0.015	2.501	0.012	0.073	0.267
**HERV9**	-0.010	0.409	0.930	0.012	2.499	0.012	0.073	0.142
**HERVH**	0.251	0.285	0.029	0.216	2.372	0.018	-0.052	0.911
**HERVFRD**	-0.064	0.474	0.562	0.029	2.086	0.037	0.510	0.041
**HML3**	-0.174	0.377	0.169	0.010	1.767	0.077	0.113	0.072
**HERVE**	-0.198	0.499	0.073	0.010	1.743	0.081	-0.062	0.749
**HERV3**	-0.090	0.308	0.403	0.061	1.565	0.117	-0.177	0.897
**HERVL**	-0.040	0.210	0.724	0.139	1.388	0.165	0.000	0.499
**HML2**	-0.159	-0.004	0.263	0.981	-1.120	0.263	-0.193	0.907
**HML6**	-0.008	0.165	0.920	0.263	1.113	0.266	0.199	0.087
**HML4**	0.105	0.091	0.438	0.564	0.963	0.335	-0.324	0.852
**MER61**	-0.053	0.151	0.664	0.374	0.694	0.487	1.077	0.014
**HML1**	-0.002	0.008	0.985	0.950	0.056	0.956	0.120	0.216

Supplementary Table S3b. Differential expression analysis of ERV families using TETranscripts in **cerebellum**, with Log2 Fold-Change, P-value from differential expression analyses, Stouffer’s meta-analysis and MAGMA ERV-set analysis using ALS GWAS. Chr: Chromosome, KCL: King’s College London, MC: Motor Cortex, FC: Fold-change

ERV Family	Log2 FC FCx GSE137810	Log2 FC PFC GSE67196	P-value FCx GSE137810	P-value PFC GSE67196	Stouffer's Meta-analysis Z	Stouffer's Meta-analysis P	MAGMA Beta	MAGMA P-value
**HERVE**	-0.168	0.473	0.028	0.003	2.077	0.038	-0.062	0.749
**PABLB**	-0.169	-0.038	0.042	0.837	-2.032	0.042	-0.021	0.525
**HML4**	0.000	-0.198	0.996	0.051	-1.951	0.051	-0.324	0.852
**HERVH**	0.040	-0.214	0.596	0.053	-1.801	0.072	-0.052	0.911
**HML5**	-0.050	0.272	0.424	0.055	1.745	0.081	0.065	0.274
**HERVI**	-0.073	-0.200	0.258	0.152	-1.735	0.083	-0.062	0.592
**HML2**	-0.006	-0.288	0.954	0.089	-1.699	0.089	-0.193	0.907
**HERV3**	-0.027	0.249	0.668	0.119	1.503	0.133	-0.177	0.897
**HERVH48**	0.000	0.198	0.995	0.190	1.312	0.190	0.196	0.186
**HML3**	-0.047	0.172	0.462	0.129	1.269	0.204	0.113	0.072
**HERV9**	-0.059	-0.087	0.371	0.405	-1.191	0.234	0.073	0.142
**HERVL**	-0.058	-0.057	0.378	0.688	-0.910	0.363	0.000	0.499
**HERVFRD**	-0.061	-0.051	0.356	0.770	-0.896	0.370	0.510	0.041
**HML6**	-0.047	-0.022	0.405	0.794	-0.863	0.388	0.199	0.087
**HERVW**	0.016	-0.053	0.797	0.726	-0.262	0.793	0.073	0.267
**HML1**	0.064	-0.082	0.375	0.548	0.076	0.939	0.120	0.216
**MER61**	0.050	-0.066	0.407	0.556	0.027	0.979	1.077	0.014

Supplementary Table S3c. Differential expression analysis of ERV families using TETranscripts in **frontal cortex**, with Log2 Fold-Change, P-value from differential expression analyses, Stouffer's meta-analysis and MAGMA ERV-set analysis using ALS GWAS. Chr: Chromosome, KCL: King's College London, FC: Fold-Change, FCx: Frontal-cortex, PFC: Prefrontal cortex

SOURCE	TERM	P-VALUE	TERM SIZE (N GENES)	QUERY SIZE (N GENES	OVERLAP SIZE (N GENES	OVERLAPPING GENES
**GO:MF**	Cytokine binding	3.96E-07	131	624	22	TNFRSF1A, IL1R1, OSMR, CD93, TNFRSF1B, IL6R, THBS1, LRRC32, ITGB3, PLP2, FZD4, PDPN, GBP1, IL13RA1, ZFP36, IL1RL1, IL18R1, CXCR1, CXCR2, IL1R2, VASN, PRLR
**GO:MF**	Cytokine receptor activity	3.84E-06	99	624	18	IL1R1, OSMR, IFNGR2, IL4R, IL6R, FZD4, IL13RA1, IL15RA, CD44, IL1RL1, IL18R1, CXCR1, CXCR2, IL1R2, IL18RAP, IL7R, PRLR, IFNLR1
**GO:MF**	Immune receptor activity	6.62E-05	131	624	19	IL1R1, OSMR, IFNGR2, IL4R, IL6R, FZD4, IL13RA1, IL15RA, CD44, IL1RL1, IL18R1, FPR2, CXCR1, CXCR2, IL1R2, IL18RAP, IL7R, PRLR, IFNLR1
**GO:MF**	Growth factor binding	1.08E-03	142	624	18	IL1R1, OSMR, CD93, SHC1, COL4A1, IL6R, THBS1, LRRC32, ITGB3, FLT1, IL13RA1, HAP1, HTRA3, IL1R2, FGFR1, KL, VASN, ITGB4
**GO:MF**	Cell adhesion molecule binding	3.44E-03	506	624	38	S1PR3, TAGLN2, CLIC1, MSN, ZC3HAV1, ICAM1, ANXA2, MMP14, BAG3, THBS1, TGFBI, PDLIM1, ITGB3, GPRC5A, CEMIP2, F11R, VASP, LYN, CNN2, ANXA1, TRIM25, GFAP, EPS8L2, SFN, CDH6, MPP7, KRT18, TRPC4, DNAJB1, AHSA1, ARHGAP18, VASN, EPHA2, HSPA1A, CD177, ITGB1BP2, ITGB4, OLFM4
**GO:BP**	Cytokine-mediated signaling	4.71E-26	799	617	99	TNFRSF1A, STAT3, CASP4, GBP2, IL1R1, OSMR, IFITM2, SHC1, BCL6, MSN, TIMP1, IFNGR2, IFITM3, CEBPD, TNFRSF1B, ICAM1, IFITM1, HGF, MCL1, CDKN1A, ANXA2, LTBR, TNFRSF11B, PIM1, IL4R, BST2, PARP9, IL6R, TRIM5, SOCS3, PTGS2, IRAK3, MYD88, TNIP2, PLP2, MAP3K8, PSMB8, FZD4, B2M, TRIM22, GBP1, CNN2, TNFAIP3, ANXA1, TAB2, TRIM25, IL13RA1, IL15RA, STAT6, NOD1, CD40, NOD2, MYC, TRIM21, CCL2, CD44, CSF1, CXCL2, IL1RL1, IL18R1, CSF3, IGHG4, SP100, NFKBIA, CXCR1, CARD16, IL6, LIF, KRT18, IL32, CXCR2, CISH, IL1R2, NMI, SEM1, IL18RAP, CASP8, FOS, CLDN18, IFI35, JUNB, TNFSF15, MUC1, TNFRSF12A, IL7R, GFI1, HSPA1A, IL15, CXCL1, MMP9, PRLR, OSM, TNFSF18, HSP90AA1, CCL4L2, SPHK1, CCL3L1, IFNLR1, IRF4

**GO:BP**	Inflammatory response	2.78E-19	780	617	87	PLSCR1, TNFRSF1A, STAT3, ANO6, CASP4, NAMPT, IL1R1, OSMR, S1PR3, BCL6, TIMP1, IFNGR2, SMAD1, TNFRSF1B, ICAM1, CEBPB, HGF, IL4R, TGM2, NFKBIZ, NFKB2, IL6R, THBS1, SOCS3, PTGS2, CALCRL, MYD88, B4GALT1, TNIP2, F11R, SERPINA3, MFHAS1, BACE2, SLC7A2, LYN, SBNO2, CHI3L1, S100A9, TNFAIP3, ANXA1, ZFP36, NOD1, CD40, VAMP8, NOD2, F2R, LYZ, CCL2, CD44, NUPR1, CSF1, CXCL2, IL1RL1, IL18R1, FPR2, S100A8, NFKBIA, C4A, LPL, C4B, IL6, MEFV, CXCR2, PTGIR, IL1R2, NMI, TREM1, SNAP23, IL18RAP, TRPV4, PTGER2, FOS, SELE, EPHA2, ZC3H12A, SCUBE1, LDLR, IL15, CXCL1, RELB, MMP9, CD96, OSM, CCL4L2, SPHK1, CCL3L1, SERPINC1
**GO:BP**	Myeloid leukocyte activation	8.49E-17	657	617	75	PLSCR1, ANO6, STOM, NAMPT, CD59, CD93, IFNGR2, TNFRSF1B, PECAM1, ANXA2, LTBR, IL4R, BST2, CMTM6, THBS1, LRG1, B4GALT1, PNP, TNIP2, CD58, B2M, SERPINA3, FES, MFHAS1, SLC7A2, LYN, SBNO2, TMBIM1, CHI3L1, CNN2, HEBP2, PLAU, S100A9, SLC2A3, VAMP8, LYZ, CD44, PLAUR, CSF1, IL1RL1, CRISPLD2, FCAR, SERPINB1, MGAM, FPR2, S100A8, RAB27A, FCGR3B, FABP5, CXCR1, IL6, HVCN1, CXCR2, MVP, SNAP23, IL18RAP, CDA, BATF2, ZC3H12A, RHOH, HSPA1A, FCN1, LDLR, IL15, JMJD6, CD177, CXCL1, RELB, MMP9, LTF, HSP90AA1, SPHK1, PLA2G10, IRF4, OLFM4
**GO:BP**	Leukocyte activation	8.96E-12	704	617	69	STAT3, ANO6, STOM, CD59, CD93, BCL6, TNFRSF1B, PECAM1, ICAM1, ANXA2, IL4R, BST2, NFKBIZ, XBP1, CMTM6, LRG1, B4GALT1, PNP, BCL3, CD58, B2M, SERPINA3, FES, LYN, SBNO2, TMBIM1, CHI3L1, CNN2, HEBP2, PLAU, S100A9, SLC2A3, ANXA1, STAT6, CD40, VAMP8, LYZ, CD44, PLAUR, IL18R1, CRISPLD2, FCAR, SERPINB1, MGAM, FPR2, S100A8, RAB27A, FCGR3B, FABP5, CXCR1, IL6, HVCN1, CXCR2, MVP, SNAP23, CDA, ZC3H12A, HLX, HSPA1A, FCN1, CD177, CXCL1, RELB, MMP9, LTF, TNFSF18, HSP90AA1, IRF4, OLFM4

**GO:BP**	Immune cell activation	1.19E-11	708	617	69	STAT3, ANO6, STOM, CD59, CD93, BCL6, TNFRSF1B, PECAM1, ICAM1, ANXA2, IL4R, BST2, NFKBIZ, XBP1, CMTM6, LRG1, B4GALT1, PNP, BCL3, CD58, B2M, SERPINA3, FES, LYN, SBNO2, TMBIM1, CHI3L1, CNN2, HEBP2, PLAU, S100A9, SLC2A3, ANXA1, STAT6, CD40, VAMP8, LYZ, CD44, PLAUR, IL18R1, CRISPLD2, FCAR, SERPINB1, MGAM, FPR2, S100A8, RAB27A, FCGR3B, FABP5, CXCR1, IL6, HVCN1, CXCR2, MVP, SNAP23, CDA, ZC3H12A, HLX, HSPA1A, FCN1, CD177, CXCL1, RELB, MMP9, LTF, TNFSF18, HSP90AA1, IRF4, OLFM4
**KEGG**	Tnf signaling pathway	4.05E-14	112	344	30	TNFRSF1A, TNFRSF1B, ICAM1, CEBPB, MMP14, CASP7, SOCS3, PTGS2, FAS, BCL3, MAP3K8, PIK3R3, TNFAIP3, TAB2, NOD2, MLKL, CCL2, CSF1, CXCL2, IL18R1, NFKBIA, IL6, LIF, CASP8, FOS, SELE, JUNB, IL15, CXCL1, MMP9
**KEGG**	Cytokine-cytokine receptor interaction	3.75E-11	292	344	44	TNFRSF1A, IL1R1, OSMR, TNFRSF10B, IFNGR2, TNFRSF1B, LTBR, TNFRSF11B, IL4R, IL6R, TNFRSF10D, FAS, TNFRSF10A, BMPR1A, IL13RA1, IL15RA, CD40, CCL2, CSF1, CXCL2, IL1RL1, IL18R1, CSF3, CXCR1, IL6, LIF, IL32, CXCR2, GDF15, IL1R2, IL18RAP, TNFSF15, BMP4, TNFRSF12A, IL7R, IL15, CXCL1, PRLR, TNFRSF10C, OSM, TNFSF18, CCL4L2, CCL3L1, IFNLR1
**KEGG**	Nf-kappa b signaling pathway	1.72E-07	100	344	21	TNFRSF1A, IL1R1, ICAM1, LTBR, NFKB2, PTGS2, MYD88, LYN, PLAU, BCL2A1, TNFAIP3, TAB2, TRIM25, GADD45B, CD40, CXCL2, NFKBIA, DDX58, CXCL1, RELB, CCL4L2
**KEGG**	Epstein-barr virus infection	3.53E-06	197	344	28	STAT3, CDK2, ICAM1, CDKN1A, NFKB2, NEDD4, TAP1, FAS, MYD88, CD58, B2M, LYN, PIK3R3, TNFAIP3, TAB2, GADD45B, CD40, GADD45A, MYC, CD44, NFKBIA, IL6, RUNX3, SEM1, CASP8, DDX58, TAP2, RELB
**KEGG**	Viral protein interaction with cytokine	4.18E-06	98	344	19	TNFRSF1A, TNFRSF10B, TNFRSF1B, LTBR, IL6R, TNFRSF10D, TNFRSF10A, CCL2, CSF1, CXCL2, IL18R1, CXCR1, IL6, CXCR2, IL18RAP, CXCL1, TNFRSF10C, CCL4L2, CCL3L1
**CLINVAR**	HIV Type 1	1.48E-03	NA	NA	NA	IL4R; CCL3L1; CXCR1; CCL2
**CLINVAR**	Lymphoproliferative syndrome	1.92E-02	NA	NA	NA	CASP8; FAS
**CLINVAR**	Schizophrenia	3.30E-02	NA	NA	NA	APOL4; SLC1A1; CHI3L1

**CLINVAR**	Hyperlipoproteinemia	3.42E-02	NA	NA	NA	LPL; LDLR
**CLINVAR**	Alzheimer's disease	5.23E-02	NA	NA	NA	NOS3; PLAU

Supplementary Table S4. Selected gene function enrichment results of yellow network-module genes (of which HML6.3p21c is a member) from the co-expression network analysis using the KCL motor cortex. Function enrichment analysis performed using g:profiler and Enrichr.

BP: Biological Process; MF: Molecular Function; KEGG: Kyoto Encyclopaedia of Genes and Genomes pathways; ClinVar: NCBI ClinVar database; P-value: Statistical significance test of gene function enrichment; Term size: Number of genes attributed to the ontological category; Query size: Number of genes from the yellow network-module relevant to the ontological category; Overlap size: Number of genes that overlap between Term size and Query size; Overlapping genes: The genes that overlap between Term size and Query size, and which are contributing to significant enrichment for the ontological category.

SOURCE	TERM	TERM ID	P-VALUE	ADJ. P-VALUE	TERM SIZE	INTERSECT
**GO:MF**	cytokine binding	GO:0019955	NA	3.96E-07	131	22
**GO:MF**	cytokine receptor activity	GO:0004896	NA	3.84E-06	99	18
**GO:MF**	immune receptor activity	GO:0140375	NA	6.62E-05	131	19
**GO:MF**	growth factor binding	GO:0019838	NA	0.001075	142	18
**GO:MF**	cell adhesion molecule binding	GO:0050839	NA	0.003438	506	38
**GO:MF**	TRAIL binding	GO:0045569	NA	0.003845	5	4
**GO:MF**	N-acetyllactosaminide beta-1,6-N-acetylglucosaminyltransferase activity	GO:0008109	NA	0.023074	3	3
**GO:MF**	interleukin-1 receptor activity	GO:0004908	NA	0.025464	7	4
**GO:MF**	ATPase regulator activity	GO:0060590	NA	0.025634	39	8
**GO:MF**	enzyme inhibitor activity	GO:0004857	NA	0.030908	379	29
**GO:BP**	cytokine-mediated signaling pathway	GO:0019221	NA	4.71E-26	799	99
**GO:BP**	inflammatory response	GO:0006954	NA	2.78E-19	780	87
**GO:BP**	myeloid leukocyte activation	GO:0002274	NA	8.49E-17	657	75
**GO:BP**	leukocyte activation involved in immune response	GO:0002366	NA	8.96E-12	704	69
**GO:BP**	cell activation involved in immune response	GO:0002263	NA	1.19E-11	708	69
**GO:BP**	response to molecule of bacterial origin	GO:0002237	NA	1.76E-11	367	47
**GO:BP**	response to lipopolysaccharide	GO:0032496	NA	4.25E-11	347	45
**GO:BP**	cytokine production	GO:0001816	NA	6.00E-11	837	75
**GO:BP**	leukocyte mediated immunity	GO:0002443	NA	9.20E-11	880	77
**GO:BP**	response to lipid	GO:0033993	NA	1.08E-10	956	81
**GO:CC**	cell surface	GO:0009986	NA	8.40E-09	918	72
**GO:CC**	specific granule	GO:0042581	NA	4.48E-08	158	25
**GO:CC**	secretory granule	GO:0030141	NA	2.29E-06	839	62
**GO:CC**	tertiary granule	GO:0070820	NA	1.17E-05	163	22
**GO:CC**	specific granule membrane	GO:0035579	NA	1.8E-05	90	16
**GO:CC**	extracellular matrix	GO:0031012	NA	0.000136	534	42
**GO:CC**	secretory granule membrane	GO:0030667	NA	0.000138	299	29
**GO:CC**	collagen-containing extracellular matrix	GO:0062023	NA	0.000463	407	34
**GO:CC**	secretory granule lumen	GO:0034774	NA	0.001554	319	28
**GO:CC**	tertiary granule membrane	GO:0070821	NA	0.001694	72	12
**KEGG**	TNF signaling pathway	KEGG:04668	NA	4.05E-14	112	30
**KEGG**	Cytokine-cytokine receptor interaction	KEGG:04060	NA	3.75E-11	292	44
**KEGG**	NF-kappa B signaling pathway	KEGG:04064	NA	1.72E-07	100	21
**KEGG**	Epstein-Barr virus infection	KEGG:05169	NA	3.53E-06	197	28
**KEGG**	Viral protein interaction with cytokine and cytokine receptor	KEGG:04061	NA	4.18E-06	98	19
**KEGG**	Osteoclast differentiation	KEGG:04380	NA	1.09E-05	125	21
**KEGG**	JAK-STAT signaling pathway	KEGG:04630	NA	1.58E-05	162	24
**KEGG**	IL-17 signaling pathway	KEGG:04657	NA	4.54E-05	92	17
**KEGG**	Fluid shear stress and atherosclerosis	KEGG:05418	NA	0.000873	137	19
**KEGG**	Pathways in cancer	KEGG:05200	NA	0.001409	529	45
**KEGG**	Human T-cell leukemia virus 1 infection	KEGG:05166	NA	8.00E-03	216	23
**REAC**	Cytokine Signaling in Immune system	1280215	NA	5.55E-18	831	93

**REAC**	Signaling by Interleukins	449147	NA	5.58E-17	459	65
**REAC**	Interleukin-4 and Interleukin-13 signaling	6785807	NA	8.34E-14	110	29
**REAC**	Interleukin-10 signaling	6783783	NA	3.41E-09	45	16
**REAC**	Neutrophil degranulation	6798695	NA	3.97E-07	476	49
**REAC**	Interferon Signaling	913531	NA	0.001245	193	23
**REAC**	Interferon gamma signaling	877300	NA	0.003809	87	14
**REAC**	Interleukin-1 family signaling	446652	NA	0.004364	138	18
**REAC**	Interleukin-6 family signaling	6783589	NA	0.014005	24	7
**REAC**	TP53 Regulates Transcription of Death Receptors and Ligands	6803211	NA	0.029398	12	5
**CLINVAR**	human immunodeficiency virus type 1, susceptibility to	NA	0.001482	0.269767	NA	NA
**CLINVAR**	atypical hemolytic uremic syndrome	NA	0.00378	0.34402	NA	NA
**CLINVAR**	hemolytic-uremic syndrome	NA	0.00378	0.229347	NA	NA
**CLINVAR**	holoprosencephaly sequence	NA	0.00525	0.238888	NA	NA
**CLINVAR**	autoimmune lymphoproliferative syndrome	NA	0.019245	0.700529	NA	NA
**CLINVAR**	postmenopausal osteoporosis	NA	0.019245	0.583774	NA	NA
**CLINVAR**	pure gonadal dysgenesis 46,xy	NA	0.019245	0.500378	NA	NA
**CLINVAR**	hypogonadism with anosmia	NA	0.019245	0.43783	NA	NA
**CLINVAR**	schizophrenia	NA	0.032968	0.66669	NA	NA
**CLINVAR**	venous thrombosis	NA	0.034171	0.621918	NA	NA

Supplementary Table S5. Expanded gene function enrichment results of yellow network-module genes (of which HML6.3p21c is a member) from the co-expression network analysis using the KCL motor cortex. Function enrichment analysis performed using g:profiler and Enrichr, which includes KEGG pathways[10–12]. BP: Biological Process; MF: Molecular Function; CC: Cell Component

Gene/ERV	Log2FC	P-value	Gene/ERV	Log2FC	P-value	Gene/ERV	Log2FC	P-value
**HML6.3p21.31c**	**0.691**	**2.29E-05**	FAM198A	0.294	0.008	SLCO4A1	0.395	0.027
ITPKB	0.373	3.14E-05	COL4A2	0.472	0.009	ANO6	0.156	0.027
ANOS1	0.544	3.97E-05	SAMD11	0.760	0.009	LAMB4	0.559	0.027
MAOB	0.381	1.47E-04	SYNM	0.263	0.009	SLC15A5	0.826	0.027
GFAP	0.749	2.37E-04	PHLDA1	0.220	0.009	GEM	0.454	0.028
CSRNP1	0.463	3.33E-04	TSPAN33	0.279	0.010	NT5DC2	0.269	0.028
ROR2	-1.018	3.71E-04	NINJ1	0.219	0.012	TMEM102	0.461	0.029
DPYSL3	0.342	4.87E-04	SULF1	0.360	0.012	ITGB1BP2	0.574	0.030
ECHDC3	0.911	0.001	CREM	0.237	0.012	IFNLR1	0.399	0.030
TNFRSF12A	0.905	0.001	SGCZ	0.288	0.013	MCL1	0.207	0.030
APOLD1	0.718	0.001	ARHGAP18	0.398	0.013	POMC	-0.570	0.030
CD44	0.966	0.001	SLC1A1	0.224	0.013	SLC2A14	-0.314	0.032
WWTR1	0.347	0.001	EPHA2	0.530	0.013	PLEKHA4	0.265	0.034
CD58	0.590	0.001	LPL	0.355	0.014	BEND4	0.291	0.036
MAFF	0.478	0.001	CFB	-0.487	0.014	TRIP10	0.354	0.036
LTF	1.382	0.001	CDC45	0.976	0.014	WNT16	0.521	0.038
NUDT4	0.301	0.002	EMP3	0.468	0.014	CCDC80	0.445	0.039
GCH1	0.604	0.002	MYL12A	0.266	0.015	TUBB6	0.273	0.039
P2RY2	0.717	0.002	TMEM176A	0.347	0.016	TMEM176B	0.373	0.040
HGF	0.623	0.003	WDR1	0.204	0.016	B2M	0.316	0.040
EMP1	0.600	0.003	KCTD12	0.313	0.016	GPR4	0.357	0.040
GPR6	0.870	0.003	CHI3L1	0.695	0.016	GBP1	0.387	0.042
LRRC55	0.365	0.003	RNF152	0.293	0.017	DCST1	-0.450	0.043
PARM1	0.297	0.003	CLIC2	0.374	0.017	CD59	0.217	0.045
AQP1	0.925	0.003	TM4SF1	0.466	0.018	FXYD3	0.278	0.047
SERPINA3	1.330	0.004	MCTP2	0.420	0.019	PLSCR1	0.345	0.048
SH3BP2	0.296	0.004	A4GALT	0.496	0.019	VWA5A	0.229	0.049
CHI3L2	0.856	0.004	AGAP10P	-1.360	0.019	FCN3	0.779	0.049
KCTD19	-0.574	0.004	LVRN	0.761	0.019	ICAM1	0.382	0.049
RASL12	0.556	0.004	OSMR	0.407	0.021			
NFKBIA	0.317	0.005	RCC1	0.336	0.022			
PIK3R3	0.299	0.005	DSE	0.232	0.022			
TRHDE	0.292	0.005	PECAM1	0.285	0.023			
TEAD3	0.525	0.006	PALLD	0.238	0.023			
IL6	1.378	0.006	CCL2	0.955	0.023			
S1PR3	0.337	0.006	YBX3	0.448	0.023			
SLC7A2	0.472	0.006	PXDC1	0.323	0.025			
S100A10	0.499	0.007	TMEM263	0.168	0.026			
IMPG2	0.303	0.008	ZFP36L2	0.245	0.026			

Supplementary Table S6. Differential expression analysis of genes comparing ALS donors with controls for the yellow co-expression network-module using the KCL cohort. HML6_3p21.31c was the most significant genomic element to associate with ALS in this network.

Log2FC: Log2 Fold-Change

SOURCE	TERM	TERM ID	P-VALUE	ADJ. P-VALUE		TERM SIZE
**GO:MF**	immune receptor activity	GO:0140375	NA	2.80E-08	131	26
**GO:MF**	cytokine binding	GO:0019955	NA	4.49E-06	131	23
**GO:MF**	cytokine receptor activity	GO:0004896	NA	2.06E-05	99	19
**GO:MF**	Toll-like receptor binding	GO:0035325	NA	0.000105	12	7
**GO:MF**	IgG binding	GO:0019864	NA	0.000686	10	6
**GO:MF**	lipid binding	GO:0008289	NA	0.000903	741	60
**GO:MF**	pattern recognition receptor activity	GO:0038187	NA	0.00127	22	8
**GO:MF**	virus receptor activity	GO:0001618	NA	0.001873	75	14
**GO:MF**	immunoglobulin binding	GO:0019865	NA	0.001874	23	8
**GO:MF**	exogenous protein binding	GO:0140272	NA	0.002207	76	14
**GO:MF**	phosphotyrosine residue binding	GO:0001784	NA	0.004053	41	10
**GO:BP**	myeloid leukocyte activation	GO:0002274	NA	7.20E-51	657	132
**GO:BP**	leukocyte mediated immunity	GO:0002443	NA	2.12E-44	880	144
**GO:BP**	cell activation involved in immune response	GO:0002263	NA	8.87E-44	708	128
**GO:BP**	leukocyte activation involved in immune response	GO:0002366	NA	2.74E-43	704	127
**GO:BP**	myeloid cell activation involved in immune response	GO:0002275	NA	3.24E-41	542	109
**GO:BP**	leukocyte degranulation	GO:0043299	NA	3.28E-41	532	108
**GO:BP**	regulated exocytosis	GO:0045055	NA	2.26E-40	793	131
**GO:BP**	myeloid leukocyte mediated immunity	GO:0002444	NA	7.70E-40	549	108
**GO:BP**	exocytosis	GO:0006887	NA	1.43E-38	907	138
**GO:BP**	cytokine production	GO:0001816	NA	2.03E-38	837	132
**GO:CC**	secretory granule	GO:0030141	NA	1.03E-27	839	115
**GO:CC**	secretory granule membrane	GO:0030667	NA	1.16E-19	299	57
**GO:CC**	tertiary granule	GO:0070820	NA	7.06E-15	163	37
**GO:CC**	lytic vacuole	GO:0000323	NA	6.65E-13	697	78
**GO:CC**	lysosome	GO:0005764	NA	6.65E-13	697	78
**GO:CC**	specific granule	GO:0042581	NA	8.02E-13	158	34
**GO:CC**	cytoplasmic vesicle membrane	GO:0030659	NA	1.43E-12	780	83
**GO:CC**	cell surface	GO:0009986	NA	1.64E-12	918	92
**GO:CC**	vesicle membrane	GO:0012506	NA	2.22E-12	801	84
**GO:CC**	tertiary granule membrane	GO:0070821	NA	3.16E-12	72	23
**KEGG**	Tuberculosis	KEGG:05152	NA	1.19E-11	175	37

**KEGG**	Osteoclast differentiation	KEGG:04380	NA	1.37E-11	125	31
**KEGG**	NOD-like receptor signaling pathway	KEGG:04621	NA	9.99E-09	177	33
**KEGG**	Leishmaniasis	KEGG:05140	NA	2.73E-07	72	19
**KEGG**	Fc gamma R-mediated phagocytosis	KEGG:04666	NA	6.85E-07	92	21
**KEGG**	B cell receptor signaling pathway	KEGG:04662	NA	1.46E-06	79	19
**KEGG**	Pertussis	KEGG:05133	NA	4.57E-06	76	18
**KEGG**	Complement and coagulation cascades	KEGG:04610	NA	5.25E-06	85	19
**KEGG**	Platelet activation	KEGG:04611	NA	3.68E-05	124	22
**KEGG**	Toll-like receptor signaling pathway	KEGG:04620	NA	0.000109	102	19
**REAC**	Neutrophil degranulation	6798695	NA	3.08E-27	175	91
**REAC**	Cytokine Signaling in Immune system	1280215	NA	1.57E-09	125	89
**REAC**	Signaling by Interleukins	449147	NA	2.76E-09	177	60
**REAC**	Toll-like Receptor Cascades	168898	NA	7.36E-09	72	31
**REAC**	Platelet activation, signaling and aggregation	76002	NA	6.19E-08	92	40
**REAC**	Diseases associated with the TLR signaling cascade	5602358	NA	2.95E-06	79	11
**REAC**	Diseases of Immune System	5260271	NA	2.95E-06	76	11
**REAC**	Adaptive Immune System	1280218	NA	3.27E-06	85	78
**REAC**	Immunoregulatory interactions between a Lymphoid and a non-Lymphoid cell	198933	NA	1.17E-05	124	29
**REAC**	Trafficking and processing of endosomal TLR	1679131	NA	1.82E-05	102	8
**CLINVAR**	chronic granulomatous disease	NA	1.67E-07	3.04E-05	NA	NA
**CLINVAR**	human immunodeficiency virus type 1, susceptibility to	NA	2.39E-04	0.02176	NA	NA
**CLINVAR**	mycobacterium tuberculosis, susceptibility to	NA	0.00817	0.496068	NA	NA
**CLINVAR**	systemic lupus erythematosus	NA	0.01087	0.494938	NA	NA
**CLINVAR**	non-hodgkin lymphoma	NA	0.01784	0.649737	NA	NA
**CLINVAR**	ischemic stroke	NA	0.01784	0.541447	NA	NA
**CLINVAR**	atrial fibrillation	NA	0.02599	0.67591	NA	NA
**CLINVAR**	congenital myopathy with fiber type disproportion	NA	0.02599	0.591422	NA	NA
**CLINVAR**	limb-girdle muscular dystrophies, autosomal dominant	NA	0.05714	1	NA	NA
**CLINVAR**	alzheimer's disease	NA	0.06938	1	NA	NA

Supplementary Table S7. Gene function enrichment results of brown network-module genes (of which HML6.3p21c is a member) from the co-expression network analysis using the GSE137810 lateral motor cortex. Function enrichment analysis performed using g:profiler and Enrichr, which includes KEGG pathways[10–12]. BP: Biological Process; MF: Molecular Function; CC: Cell Component

Symbol	***Log2 Fold-Change***	***Log2FC Stand. Error***	***P-value***	***IHW Adjusted p-value***
HML6_3p21.31c	**0.691**	**0.163**	**2.29E-05**	**0.038**
CCR3	-0.180	0.188	0.339	0.854
CCR1	0.131	0.327	0.689	1
CCR2	0.549	0.517	0.289	1
CCR5	0.381	0.439	0.386	1
CCRL2	0.412	0.322	0.201	1
LTF	1.382	0.435	0.001	0.283
RTP3	0.214	0.819	0.793	1

Supplementary Table S8. Differential expression between ALS and controls for genes proximal to HML6_3p21.31c ERV in the KCL motor cortex dataset. IHW: Independent Hypothesis Weighting. Log2FC: Log2 Fold-Change

		ERV Direction	*N. Ervs*	*Beta*	*SD*	*SE*	*2018 GWAS P-value*	*2016 GWAS Meta P-value*	*2016 GWAS LMM P-value*
KCL	**Prim. MC**	**Up**	**29**	**0.315**	**0.032**	**0.178**	**0.039**	**0.043**	**0.074**
		Down	42	0.066	0.008	0.150	0.331	0.743	0.584
GSE137810	**Lat. MC**	Up	75	0.010	0.002	0.113	0.465	0.447	0.235
		Down	39	0.002	0.000	0.164	0.496	0.865	0.725
	**Med. MC**	Up	132	0.087	0.018	0.089	0.165	0.217	0.118
		Down	37	0.062	0.007	0.153	0.342	0.541	0.677
	**Cere.**	Up	120	0.073	0.015	0.093	0.216	0.274	0.228
		Down	50	-0.180	-0.024	0.149	0.888	0.995	0.999
GSE67196	**Cere.**	Up	126	0.129	0.027	0.088	0.070	0.187	0.208
		Down	3	0.444	0.014	0.739	0.274	0.127	0.318

Supplementary Table S9. Results from the ERV-set MAGMA analysis using the ALS GWAS summary datasets. Beta, standard deviation and errors reported from the 2018 ALS GWAS. Last two columns taken from the 2016 ALS linear mixed model and meta-analysis GWAS. ERVs that show increased expression in post-mortem ALS (with an association p-value < 0.05), compared to controls, show a marginal enrichment of SNPs that modify ALS risk across 3 GWAS. Note that there are overlapping samples between the three GWAS. Prim. MC: Primary Motor Cortex, Lat MC: Lateral Motor Cortex, Med. MC: Medial Motor Cortex, Cere: Cerebellum. N. Ervs: Number of ERV loci tested. SD: Beta standard deviation. SE: Standard Error. 2018 GWAS: Nicolas et al. 2016 GWAS: van Rheenen and Shatunov et al. 2016.
